# Magnetotactic *Bdellovibrionota* from a ferruginous spring

**DOI:** 10.1093/ismeco/ycag116

**Published:** 2026-04-24

**Authors:** Marine Bergot, Christopher T Lefevre, Denis S Grouzdev, Nicolas Menguy, Philippe Ortet, Yann Denis, Eric Viollier, Didier Jézéquel, Caroline L Monteil

**Affiliations:** Aix-Marseille Université, CNRS, CEA, BIAM, UMR7265 Institut de Biosciences and Biotechnologies d’Aix-Marseille, Cadarache research centre, F-13115 Saint-Paul-lez-Durance, France; Aix-Marseille Université, CNRS, CEA, BIAM, UMR7265 Institut de Biosciences and Biotechnologies d’Aix-Marseille, Cadarache research centre, F-13115 Saint-Paul-lez-Durance, France; School of Marine and Atmospheric Sciences, Stony Brook University, Stony Brook, NY 11794-5000, United States; Sorbonne Université, Muséum National d’Histoire Naturelle, UMR CNRS 7590, Institut de Minéralogie, de Physique des Matériaux et de Cosmochimie (IMPMC), 4 Place Jussieu, 75005 Paris, France; Aix-Marseille Université, CNRS, CEA, BIAM, UMR7265 Institut de Biosciences and Biotechnologies d’Aix-Marseille, Cadarache research centre, F-13115 Saint-Paul-lez-Durance, France; Plateforme Transcriptomique, Aix-Marseille Université, CNRS, IMM—FR3479, Marseille, France; Laboratoire des Sciences du Climat et de l’Environnement, LSCE–IPSL, CEA–CNRS–UVSQ–Université Paris-Saclay, 91198, Gif-sur-Yvette, France; Université Paris Cité, Institut de Physique du Globe de Paris, CNRS, Paris F-75005, France; UMR CARRTEL, INRAE & Université Savoie Mont Blanc, Thonon-les-Bains 74200, France; Aix-Marseille Université, CNRS, CEA, BIAM, UMR7265 Institut de Biosciences and Biotechnologies d’Aix-Marseille, Cadarache research centre, F-13115 Saint-Paul-lez-Durance, France

**Keywords:** magnetotaxis, biomineralization, magnetosomes, predatory bacteria, ferruginous springs

## Abstract

Magnetotactic bacteria form a highly diverse group of microorganisms, yet early exploration of their diversity was largely centered on the *Pseudomonadota*. More recently, metagenomic studies have revealed that magnetotaxis, a form of chemotaxis guided by Earth’s magnetic field, is widespread in other deep-branching phyla for which little to no ecological or biological information is available beyond that inferred from their genomes. For most of them, the morphology, ultrastructure and magnetosome chain characteristics responsible for the magnetic guidance remain unknown. While screening extreme environments for novel magnetotactic species, we observed magnetotactic *Bdellovibrionota* in the anoxic and ferruginous sediments of the Fontaine Goyon spring (France). We characterized their cell morphology and ultrastructure using magnetic enrichment, a single-cell sorting approach, and high-resolution electron microscopy. Cells display the morphology typical of the few predatory bacteria described in this phylum, and biomineralize, on average, five irregularly faceted, bullet-shaped magnetite magnetosomes along the concave side of the cell. Metagenomic analysis of approximately 100 cells revealed a potentially predatory and heterotrophic lifestyle adapted to low-O_2_ conditions. It also suggests a flexible respiratory metabolism under varying redox conditions, using iron as an alternative terminal electron acceptor. Exploring the diversity of *Bdellovibrionota* in public databases, we found 21 metagenome-assembled-genomes containing magnetosome genes. None of them harbor the canonical *mamK* actin-like gene implicated in aligning magnetosomes in described magnetotactic models. Affiliated to an undescribed class, we propose a classification scheme for the magnetotactic *Bdellovibrionota* species representing the class *Bdellonasia* class nov., for which no species had been formally described.

## Introduction

Microbial biomineralization is the process by which microorganisms facilitate the formation of minerals, often as a byproduct of their metabolic activities. This natural phenomenon plays a critical role in Earth’s biogeochemical cycles, influencing the formation and transformation of minerals in soils, sediments, and aquatic environments. It can result in diverse mineral types, such as carbonates, sulfides, and oxides, which contribute to processes like carbon sequestration, metal cycling, and the preservation of microbial life in the geological record [1, 2]. Some of these biominerals can be formed intracellularly under a genetic control. This is the case of magnetic biomineralization whereby magnetotactic bacteria (MTB) produce nanoscale, membrane-bound crystals of magnetic minerals, such as magnetite (Fe_3_O_4_) or greigite (Fe_3_S_4_) into organelles called magnetosomes [3–5]. They use them to align with the Earth’s magnetic field lines and navigate to optimal redox niches, such as low-oxygen or oxygen-depleted zones in sediments [6] or water columns [7]. MTB are very diverse in term of their phylogenetic distribution, their morphology, their metabolism and the aquatic habitats where they are found [4].

Most diversity studies conducted over the past decades have revealed a polyphyletic distribution of MTB species across 17 phyla [8]. However, most of this knowledge is based on the detection of a specific repertoire of genes encoding the magnetosome formation and positioning into metagenome-assembled genomes (MAGs) [9]. In consequence, the morphology, ultrastructure, and magnetosome chain characteristics remain unknown for most of the diversity described to date, especially in deep-branching phyla. Yet, assessing phenotypic diversity is essential for understanding not only MTB evolution, but also Earth’s early history as magnetic biomineralization provides evidence of microbial activity in ancient environments and can help reconstructing paleoenvironments through the study of preserved magnetofossils [3]. Indeed, magnetosomes could have emerged as early as microbial life itself (~3.8 billion years ago) and be associated to anaerobic metabolisms during the Archean Eon (4.0–2.5 billion years ago) [10, 11]. These intracellular minerals could have been an early adaptation of microorganisms to environmental pressures in the ancient Earth’s anoxic, ferruginous, and sulfur-rich environments [12, 13] and be preserved in the geological records upon cell death. Although the absence of bacterial fossils impedes the possibility to fully state about the processes driving magnetosome emergence, increasing our knowledge of MTB ultrastructure diversity in different ecosystems may progressively fill this gap and refine predictions.

In the primitive Earth where early life and the magnetosome emerged, ferruginous environments were a defining feature of Earth’s primitive oceans and played a central role in shaping early geochemistry and supporting the emergence of life [14, 15]. The atmosphere remained reduced, dominated by CO_2_, CH_4_, and nitrogen (N₂) [16]. Oxygen was largely absent, except in localized areas around photosynthetic microbes. Oceans were rich in dissolved iron (i.e. ferruginous) due to the lack of oxygen, which prevented the precipitation of iron oxides [12, 13]. In contrast, ferruginous aquatic habitats in this contemporary era are relatively rare compared to other types of aquatic environments. The high level of oxygen strongly affects the solubility of minerals and oxidation state of many elements, resulting in geochemical conditions that differ markedly from those of the Archean oceans [16, 17]. Both however, share similar features such as the presence of dissolved CO_2_ and high levels of dissolved iron. In natural ferruginous springs, high levels of dissolved iron form iron oxides when oxidized at emergence, giving the water a reddish color and large rust-like deposits. These springs are often located in areas with abundant iron-rich bedrock, such as regions with sedimentary or volcanic rock formations [17, 18]. This is the case of Fontaine Goyon (FG), a mineral-rich spring located northeast near the volcanic Lake Pavin in France [19]. This spring is characterized by its magmatic CO_2_ content making up almost 100% of the gas phase with a flux ~1.3 l min^−1^, whereas the water flow is ~5.6 l min^−1^ [19]. The high iron input creates an acidic reddish hue of iron oxides above the sediments of surrounding superficial waters from the Couze Pavin.

Exploring new MTB diversity in extreme environments, we sampled this spring regularly between 2015 and 2023 and systematically performed magnetic enrichment. We reproducibly observed the presence of tiny magnetotactic microorganisms almost imperceptible under the optical microscope. We then applied a single-cell sorting approach to characterize the ultrastructure and genome of these magnetically sorted populations systematically retrieved from the anoxic zone of the sediments. They were affiliated with the phylum *Bdellovibrionota* and had a unique shape, number, and organization of magnetosomes. The genome of representative species contained genes coding for a predatory system and proteins likely associated with iron-reduction. By seeking for genomic traces of magnetotaxis in this phylum more largely, we found that several published MAGs in this group could be magnetotactic and predatory bacteria, but not all are iron reducing and may not all be associated to ferruginous habitats. Finally, we propose a classification scheme for the undescribed class to which magnetotactic *Bdellovibrionota* were affiliated with and for which no species had been described before.

## Material and methods

### Samples collection

FG spring is a ferruginous soda spring located northeast near a volcanic Lake Pavin in Auvergne, Massif Central, France (45.509391°N, 2.904215°E) at an elevation of 1085 m above sea level [19]. As the iron-rich and acidic water reaches the surface and oxidizes, it forms reddish iron oxide deposits overlain by anoxic water (Fig. S1). Samples were repeatedly collected in spring and autumn from 2015 to 2023. Onshore sampling was done by fully filling one-liter glass bottles with ~500 ml of sediments and ~500 ml of water overlying the sediments. Air bubbles were excluded. Once in the laboratory, mesocosms were stored with their cap closed, in the dark at 4°C. Some of these mesocosms were analyzed 1 h after collection whereas others were stored at 4°C in the dark for 7 days.

### Physicochemical characterization of the spring

Water was sampled from the channel continuously fed by the FG spring above the sediment where MTB were found (Fig. S1 and Table 1). *In situ* measurements (i.e. temperature, electrical conductivity of water measured at 25°C, dissolved oxygen, and pH) were performed with a WTW multi-parameter portable meter Multi 3620 IDS. Laboratory chemical analyses were performed by Inductively Coupled Plasma—Atomic Emission Spectroscopy (ICP-AES) (ICAP 6000 ThermoFisher Scientific) for major and minor elements, by Ionic Chromatography (Dionex) for major anions, and by immediate spectrophotometric measurements for alkalinity [20] and total dissolved sulfide [21]. Dissolved oxygen concentration was measured as described previously (Supplementary Methods S1) [22].

**Table 1:** Physico-chemical characterization of the FG spring (July 2021) Physico-chemical characterization of FG Spring. “–” denotes cases where no analysis was conducted.

	Value	
Variable	This study (July 2021)	Assayag *et al.* [19]
Date of sampling and *in situ* measurement	13/07/2021	13/04/2004
T (°C)	8.6	4.9
C25 (μS.cm^−1^)	994	1038
Salinity (psu)	0.4	–
pH	5.61	4.5
O_2_ (%)	0.6	–
O_2_ (mg/L)	0.06	–
Alkalinity (μM)^a^	11 651	–
PO_4_^3−^ (μM)^b^	11.3	11.5
Fe (μM)^b^	527	534
Mn (μM)^b^	44.4	43.3
SiO_2_ (μM)^b^	1414	–
Na (μM)^b^	610	744
K (μM)^b^	275	286
Mg (μM)^b^	2467	2407
Ca (μM)^b^	2510	2636
Cl^−^ (μM)^c^	95	100
SO_4_^2−^ (μM)^c^	2.7	15–30
NO_3−_	0	–
H_2_S total	0	–
Sr (μM)^b^	4.12	4.2
Ba (μM)^b^	2.8	3
Al (μM)^b^	1.02	–
Cations total	11 994	–
Anions total	11 763	–
IB %^d^	2	–
Ionic force (mol.L^−1^)	0.0174	–
Calculated C25 (μS.cm^−1^)	989	–
Delta C25 measured / calculated	−0.50%	–
HCO_3_^−^ (μmol.KgSW^−1^)	11 652	–
CO_3_^2−^ (μmol.KgSW^−1^)	0.638	–
CO_2_ (μmol.KgSW^−1^)	74 796	–
*p*CO2 (atm)^e^	1,34	–
DIC (mM)	86	80

a Measured by colorymetric methods.

^b^Measured by ICP-AES.

^c^Measured by ionic chromatography.

^d^Ionic balance = (sum Cations and Anions / average Cations and Anions) x100.

^e^Calculated with the CO_2_Sys model.

#### Magnetic enrichments and light microscope observations

North-seeking magnetotactic cells were concentrated by placing the south pole of a bar magnet for 1 h next to the bottles and above the sediment–water interface. Pellets of magnetotactic cells aggregated against the magnets were harvested and exanimated using the hanging drop technique [23] under a Zeiss Primo Star light microscope equipped with phase-contrast and differential interference contrast optics. In this technique, water and sediment containing MTB are deposited onto a coverslip. The coverslip is then inverted and positioned within a rubber O-ring on an optical microscope slide. A magnet is placed near the drop for 10 min to direct the magnetotactic cells toward one edge. Magnetotaxis is then confirmed by rotating a stirring-bar magnet 180° on the microscope stage, which reverses the local magnetic field and triggers a directional response from the cells.

#### Cell sorting and whole genome amplification

Cell sorting was carried out on sediment samples as described previously (Supplementary Methods S2) [22]. To obtain sufficient gDNA for 16S rRNA gene and shotgun metagenomic sequencing, whole genome amplification (WGA) was carried out using the multiple displacement amplification technique with the REPLI-g Single Cell Kit (QIAGEN) following the manufacturer’s instructions. Because the MTB cells were very small and nearly indistinguishable under the optical microscope, WGA was performed on a bulk sample containing about 10 to 100 cells sorted with a micromanipulator. The 16S rRNA gene sequence was amplified from several DNA samples and used to design FISH probes as described below. Only one of the DNA samples was used for metagenome sequencing. The concentration of double strand gDNA was measured using a QuBit™ 4 fluorimeter (ThermoFisher Scientific) before sequencing.

#### Cloning and sequencing of the 16S rRNA genes

The 16S rRNA gene of DNA samples was amplified using the Phusion® Hot Start Flex DNA Polymerase following the manufacturer’s recommendations, a DNA template of 50–70 ng/μL, and the 27F 5′-AGAGTTTGATCMTGGCTCAG-3′ and 1492R 5′-TACGGHTACCTTGTTAC-GACTT-3′ primers. Blunt-end fragments of 16S rRNA gene sequences were cloned using a Zero Blunt® TOPO® PCR Cloning Kit with One Shot® TOP10 chemically competent *Escherichia coli* cells. The inserts of 14 resulting clones were sent for pyrosequencing (Eurofins Genomics Germany GmbH). Sequences were clustered into two operational taxonomic units (OTUs) and then compared to sequences from the NCBI nucleotide database with the BLASTN algorithm [24, 25]. The 16S rRNA gene sequences of the sorted MTB OTUs were checked using the UCHIME2 chimera detection algorithm [26].

#### Transmission electron microscopy

Magnetotactic cells of several samples were aggregated at the edge of the northern side of a hanging drop. Two microliters containing magnetotactic cells were pipetted at the edge of a hanging drop and were carefully deposited onto the center of a transmission electron microscopy (TEM) grid pre-coated with poly-L-lysine and covered with a carbon film. As the drop began to dry, the grid was gently rinsed with filtered deionized water.

Electron micrographs were recorded with a Tecnai G^2^ BioTWIN (FEI Company) equipped with a charge-coupled-device (CCD) camera (Megaview III, Olympus Soft imaging Solutions GmbH) using an accelerating voltage of 100 kV. High-resolution transmission microscopy (HRTEM) was carried out using a JEOL 2100F microscope. This machine, operating at 200 kV, was equipped with a Schottky emission gun and an ultrahigh resolution pole piece. HRTEM images were obtained with a Gatan US4000 CCD camera.

Images obtained using light and electron microscopy were used to measure cells and magnetosomes size and shape factor (length/width) using the ImageJ software version 1.53 (https://imagej.net/ij/). Several cells were used to estimate an average and a standard deviation using the R software v4.1.2 [27]. The mineralogical identification of the magnetosomes was achieved using Fast Fourier Transform (FFT) analysis, which converts real-space images into frequency space and generates diffraction patterns that allow measurement of interplanar spacings and indexing of crystal lattices.

#### Fluorescence *in situ* hybridization

Fluorescence *in situ* hybridization (FISH) was performed according to the procedure described previously [28] using a Zeiss LSM 980 confocal microscope with a 63×/1.4 NA oil immersion lens. An ATTO488-labeled probe specific to the 16S rRNA gene sequences obtained from FG was designed: FGp, 5′-ATTO488-TTGCGCTTTCGCTTCCCTCTGTACCGACCA-3′. The probe sequence corresponds to a 30 pb-long region complementary to nucleotides 1243 to 1272 of the 16S rRNA molecule with at least one or two mismatches with non-target *Pseudomonadati* species (i.e. NCBI identifiers AF418177.1; AF418179.1; M26632.1; AF373920.1; X80725.1; L07834.1). The probe specificity was checked by aligning its sequence against all 16S rRNA gene sequences deposited in the public NCBI “Core nucleotide database” in March 2026 using the Basic Local Alignment Search Tool (BLAST) with default settings, but was also evaluated by using the PROBE_MATCH program in the RDP-II [29]. Oligonucleotide probes used in this study were purchased from Eurofins Genomics.

#### Metagenome sequencing and assembling

Whole-genome sequencing was performed on the amplified DNA of a sample containing about 100 cells sorted with a micromanipulator. DNA libraries were prepared from the fragmented DNA using the Nextera XT DNA Library Preparation Kit from Illumina®, according to the manufacturer’s instructions. Paired-end (2 × 150 bp) DNA sequencing was performed on the MiSeq sequencer (Illumina®, San Diego, CA, USA) hosted at the IMM Transcriptomic and Genomic facility with a MiSeq v2 (300-cycles) flow cell (Illumina®, San Diego, CA, USA). Raw data were processed with Trimmomatic [30] with default settings, leading to a total of 3 × 10^6^ paired-end high quality reads. An assembly was launched using SPAdes version 3.9.0 with k-mer sizes 21,33,55,77,99, and 127, using the options --only-assembler, --sc, and --careful. The mini-metagenome assembled from the amplified genome of ~100 magnetically sorted cells was processed following the “Anvi’o User Tutorial for Metagenomic Workflow” to visualize them (Supplementary S3) [31]. In summary, contigs are automatically compared and clustered by their sequence composition (i.e. tetranucleotide frequencies), which tend to be similar within a genome. Genome bins are manually refined in the Anvi’o interface using hierarchical clustering visualizations and taxonomy annotations. This procedure led to the reconstruction of two MAGs named FG-1 and FG-2.

#### Genome-based taxonomic classification and molecular phylogeny

First, GTDB-Tk v2.1.1 [32] was used to assign each MAG to a taxonomic group based on the Genome Taxonomy Database (GTDB) classification release 220 [33] (https://gtdb.ecogenomic.org). Then, phylogenetic trees were built using whole genomes as previously described (Supplementary S4) [22]. In brief, we used the maximum likelihood method implemented in the IQ-TREE v2.2 software [34, 35] and a concatenated alignment after removing outlier genes with PhylteR [36]. Pairwise average amino acid identity (AAI) percentages between FG MAGs and closest relatives were estimated using the compareM tool (https://github.com/dparks1134/CompareM) with default parameters.

#### Functional annotation and metabolic pathways prediction

MAGs were analyzed using the MicroScope platform [37] for further comparative analysis of the magnetosome gene clusters (MGCs) and expert functional annotation. Metabolic pathways were inferred using the MetaCyc database v 27.0 [38] and a workflow using Pathway Tools [39] and KoFamScan [40] as previously described (Supplementary S5) [22]. A third approach using FeGenie [41], AlphaFold [42], and Foldseek [43] was also applied specifically to search for molecular determinants of dissimilatory iron reduction and oxidation (Supplementary S6).

## Results

### Magnetotactic bacteria from the ferruginous Fontaine Goyon spring are represented by a single morphotype only

The diversity of MTB populations was assessed in the reddish iron oxide deposits of the FG spring. A geochemical characterization of the water and sediments was conducted on 13 July 2021 and compared with that of March 2004 [19] (Table 1), to link the diversity and physiology of MTB to their environmental context. While the conductivity remained as high (i.e. 994 μS.cm^−1^ versus 1038 μS.cm^−1^), the pH was less acid than 20 years ago (i.e. 5.6 versus 4.5). Not measured previously [19], the *p*CO_2_ was 1.34 atm in 2021, which is several folds above the values encountered in most freshwater systems [44]. The water chemistry was typical of such an environment, with an iron concentration of 527 μM. Sulfate concentration was low (<2.7 μM) and nitrate was undetectable. *In situ* oxygen concentration measurements indicated that the water coming out of the spring and above the sediment contained <0.6 mg.l^−1^ of dioxygen. This low level persisted below the sediment–water interface.

One-liter bottles containing a 50/50 (vol/vol) mixture of sediment and water were collected and stored at room temperature with the cap closed prior to magnetic concentration. Although samples could be slightly oxygenated during collection, measurements of O_2_ over days indicated that they rapidly returned to complete anoxia from Day 1. MTB concentration was measured by positioning a magnetic bar next to 20-μL hanging drops sampled at different depths of the mesocosms. Whatever the sample, sediments of the FG spring contained abundant north-seeking MTB that accumulated at the edge of the hanging drop under the light microscope. They were thriving below the sediment–water interface in complete anoxia. Although an exact count was not possible due to the small size of the cells and the abundance of fine sediment particles surrounding them, we estimated a concentration of approximately 10^4^ to 10^5^ MTB cells per milliliter of pore water.

**Figure 1:**
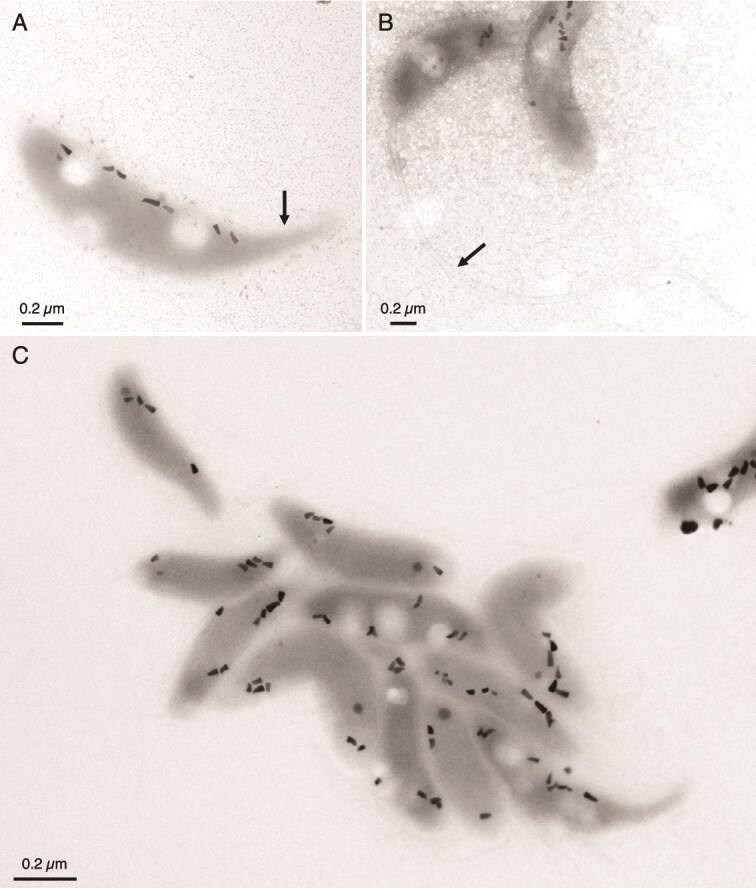
Transmission electron microscope images of magnetotactic vibroid bacteria from the FG spring. Panel (A) highlights the pointed end of a cell containing magnetosomes, indicated by a arrow. Panel (B) emphasizes the thick flagellum of another magnetotactic vibrio. Panel (C) shows several magnetotactic bacteria sticked together. Additional pictures are given in Fig. S2.

At a first glance cells escaped our observation due to their small size and low contrast under the light microscope, even using the phase contrast. They were observable through an ×40 objective when cells were abundant, as a contrasting, vibrant wave, but with indistinguishable cells. Individual cells could be observed with at least a ×63 objective only, and their shape and size could be determined solely from TEM images (Fig. 1A and Fig. S2). Cells were vibrioid with an average length and width of 1.45 ± 0.17 μm and 0.40 ± 0.04 μm (*n* = 30), respectively. A single flagellum was observed at one pole of the cells (Fig. 1B). This flagellum was thick (i.e. ⁓53.2 ± 6.6 nm) compared to the size of the cells reminiscent of that commonly observed in predatory bacteria such as *Bdellovibrio* spp. [45]. Another characteristic of the cells is the presence of a 200–300 nm-long, sharp, asymmetrical, elongated, and bottlenose-shaped anterior pole, at the pole opposite to the flagellum (Fig. 1A).

**Figure 2:**
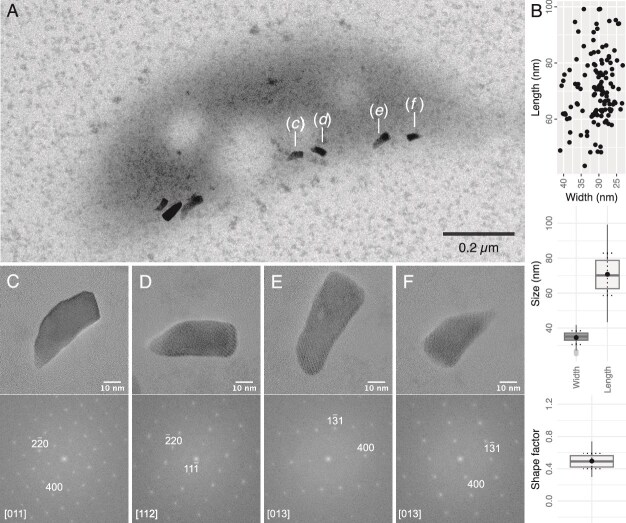
Characteristics of magnetosomes observed in magnetotactic bacteria from the FG spring. (A) TEM image of a magnetotactic bacterium isolated from FG spring. (B) Distribution of magnetosome crystals for cells isolated from FG spring, with their average length, width and shape factor (*n* = 126). (C–F) high-resolution (HR) TEM images of the crystals annotated in (A) and corresponding FFT patterns indexed with the magnetite structure (space group *fm3̅m*, with *a* = 8.04 Å, *d*_111_ = 4.85 Å, *d*_220_ = 2.97 Å, *d*_113_ = 2.53 Å and *d*_400_ = 2.10 Å) representing the magnetosomes features observed in FG MTB. Reference from the international crystal structure database for magnetite is ICSD-65339.

A single chain of 5 ± 2 (*n* = 62) bullet-shaped particles measuring 70.8 ± 12.1 nm in length and 34.2 ± 4.2 nm in width was systematically observed against the concave side of the cells (Fig. 1; Fig. S2 and Fig. 2A). The length, width, and shape factor distributions (Fig. 2B) resemble to those of bullet-shaped magnetosomes generally found in MTB of the *Desulfobacterota* and *Nitrospirota* taxa [46]. However, crystal seem to be irregularly faceted and often flattened on two sides. HRTEM observations indicate that all crystals found in bacteria of FG are magnetite (Fig. 2C–F). Although apparently aligned, magnetosomes do not form a plain chain and are sometimes spaced by low contrasted globules under the TEM.

### Magnetotactic bacteria from the Fontaine Goyon spring are affiliated with the phylum *Bdellovibrionota*

Because of their size, individual single cells could not be sorted with our equipment, which is designed to sort bacteria under the light microscope. Therefore, a gDNA amplification was carried out on 10–100 cells from homogeneous MTB populations. Amplification of the cloned 16S rRNA gene from the amplified DNA produced two OTU sequences sharing 94.1% identity. Based on sequence identity thresholds used to set taxonomic boundaries [47], MTB from FG were composed of different species, likely belonging to two genera. Based on BLASTN alignments (March 2026), the closest cultured species shared low sequence identities with MTB from FG (i.e. between 84.6% and 85.0% on ~1500 pb) and belong to the kingdom *Pseudomonadati* [48], such as *Anaeromyxobacter*, *Geobacter*, *Syntrophotalea*, and *Desulfuromonas* genera. However, such sequence identities are considered poorly predictive for taxonomic affiliation, and additional genomic analyses are required to infer evolutionary relationships. FISH experiments using specific fluorescently labelled oligonucleotide probes were designed using the two sequences and further validated that the amplified DNA corresponds to the MTB morphotype observed under light and transmission electron microscopy (Fig. S3).

Genomic DNA of the same sample was further used to sequence the whole genomes of MTB populations from FG spring. Two MAGs were recovered from the assembly: one high-quality MAG 97.6% complete and 4.91% redundant, and one medium-quality MAG 84.8% complete and 4.41% redundant [49]. Only the first MAG harbors a 16S rRNA gene sequence, identical to one of the OTUs obtained by pyrosequencing. These two MAGs named FG-1 and FG-2 (Table S1), were positioned in the tree of *Bacteria*. A GTDB-tk analysis [32] affiliated both MAGs with a family without official name in the nomenclature, named UBA1018, within the order and class of the same name within the phylum *Bdellovibrionota.* In GTDB release 220, this phylum *Bdellovibrionota* comprises 11 classes, of which three currently have an official status in the List of Prokaryotic names with Standing in Nomenclature (LPSN) (https://lpsn.dsmz.de/): the *Bacteriovoracia*, *Bdellovibrionia*, and *Oligoflexia*. In the more recent release R226, two of the most basal classes (i.e. UBA2361 and *Oligoflexia*) were excluded from the phylum and together form the distinct phylum Bdellovibrionota_B*.*

**Figure 3:**
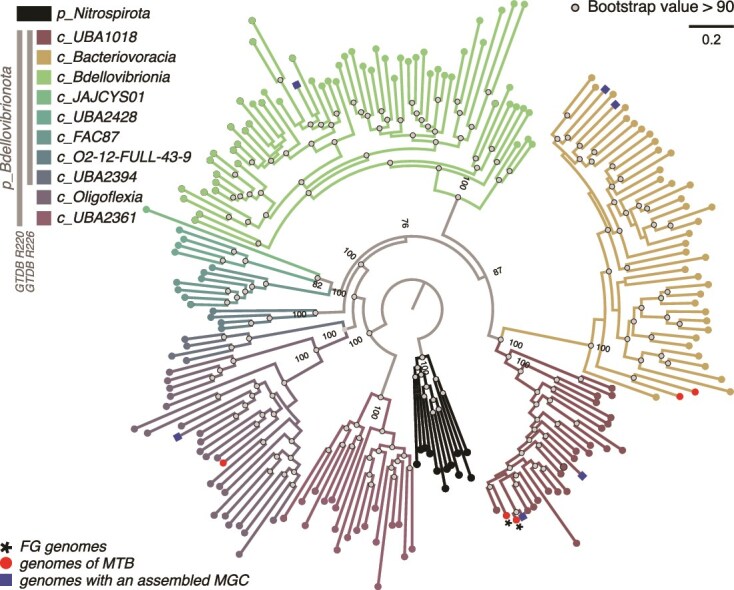
Maximum-likelihood tree of the phylum *Bdellovibrionota.* The tree was built based on 120 conserved bacterial markers used in GTDB [33] and shows the distribution of FG MAGs, magnetotactic bacteria and MAGs with an assembled MGC. Each *Bdellovibrionota* genus in the GTDB R220 with at least one genome of good quality (i.e. > 90% complete with <5% redundancy according to CheckM2 [49]) is represented by one genome leading to a set of 193 genomes. It was rooted with representative members of the phylum *Nitrospirota*. Colors represent classes in GTDB release 220 (https://gtdb.ecogenomic.org). In the GTDB release 226, the phylum *Bdellovibrionota* is split into two, with the *Oligoflexia* and UBA2361 classes being reassigned into an undescribed phylum: The phylum Bdellovibrionota_B. Genomes representing GTDB genera in which MTB were affiliated to in this study and previously [9] were colored in red. Those highlighted in blue represent GTDB genera in which we identified at least one MAG containing an MGC, derived from a metagenome without magnetic purification. A complete version of this tree is given Fig. S4 and Data S1 (Newick format). Branch lengths represent the number of substitutions per site. Tree topology was tested using an ultrafast bootstrap approximation approach with 1000 replicates [52]. The gray circles represent bootstrap values >90%. The values next to some of the internal nodes represent the statistical support (ultra-fast bootstrapping approach 1000 replicates).

**Figure 4:**
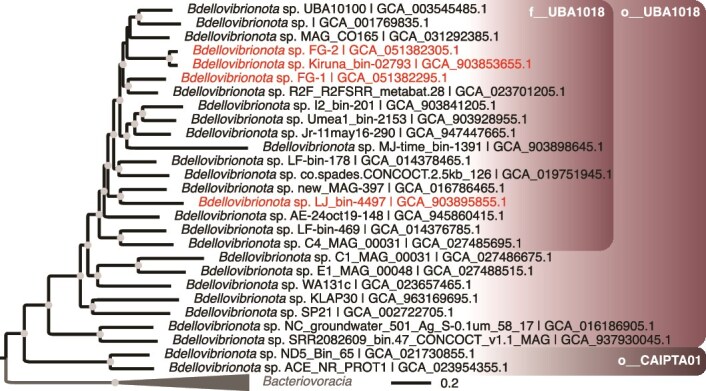
Maximum-likelihood tree of the class UBA1018. The tree was built based on 120 conserved bacterial markers used for the GTDB [33] and shows the fine relationships between FG MAGs and high-quality genomes representing each species. We selected a set of genomes of good quality (i.e. > 90% complete with <5% redundancy according to CheckM2 [49] representing each genus (February 2025). The tree was rooted with representative members of the phylum *Bacteriovoracia*. Branch lengths represent the number of substitutions per site. Tree topology was tested using an ultrafast bootstrap approximation approach with 1000 replicates [52]. The gray circles represent bootstrap values >90%. The corresponding GenBank accession numbers are given in the sequence names. The two orders of the class c__UBA1018, along with the family f__UBA1018 proposed in GTDB release 220 [33], to which the FG MAGs belong, are mapped onto the tree. The red names indicate genomes with an assembled MGC. An extended version with a representative genome per species and a complete taxonomy is given in Data S2 (Newick format).

A maximum likelihood tree was built to resolve evolutionary relationships between these classes with 191 high-quality genomes representing each genus of the phylum, using 120 conserved markers and the model LG + I + I + R10 as the substitution model (Fig. 3, Fig. S4 and Data S1). This tree challenges the classification in GTDB release 226, as the phylum *Bdellovibrionota_B* is paraphyletic. The class UBA1018 to which FG MAGs are affiliated with, is a sister clade to the *Bacteriovoracia*. Together with the *Bdellovibrionia,* they form the most recent groups of the *Bdellovibrionota* (Fig. 3)*.* We built a phylogenic tree based on the same conserved genes to explore further the genetic relationships with other members of the class UBA1018 (Fig. 4 and Data S2). Although closely related, FG MAGs do not form a monophyletic group (Fig. 4). Whereas FG-2 represents a novel species clustering with genomes affiliated with a published undescribed genus CAIRTR01, the FG-1 taxonomic position is different. Although GTDB-tk classified the FG-1 species within the same genus, our phylogenetic reconstruction and pairwise AAI analysis showed that this MAG is the only representative of an undescribed species and genus close to the genus JAMPXJ01 with an average AAI of 62% (i.e. MAG named R2F R2FSRR metabat.28; Data S2 and Fig. S5). Indeed, their AAI percentage values are all below the 65% threshold recommended to define the genus boundary [50,51].

### Magnetotaxis is polyphyletically distributed in *Bdellovibrionota*

**Figure 5:**
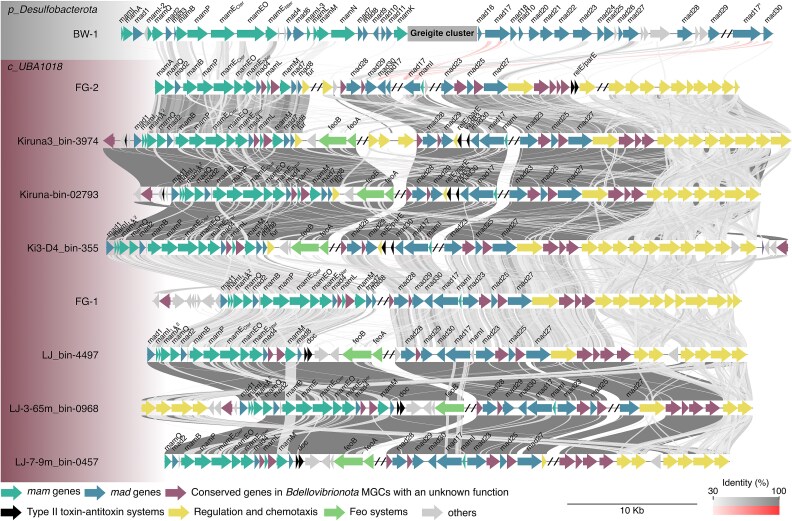
Conservation of MGC synteny in the FG MAGs and the other *Bdellovibrionota* MAGs of the class UBA1018. Each arrow represents a gene. Homologous families were then determined by the presence of conserved domains using the microscope platform [37]. The annotated magnetosome genes described in the reference genomes of *Magnetospirillum gryphiswaldense* MSR-1 [55] and *Desulfamplus magnetovallimortis* BW-1 [53] are colored in turquoise and blue (i.e. *mam* and *mad* genes, respectively). Genes of unknown function or not conserved in MTB are colored in grey whereas those conserved in magnetotactic *Bdellovibrionota* only, are colored in wine red. Slashes represent mainly truncations and sometimes regions spacing two putative operons. Sequence identities were estimated with BLASTP alignments and are represented by bands, with their intensity reflecting the percentage of identity. Homologous regions in two genomes are connected by grey or red links if they are oriented in the same or opposite direction, respectively. The MGC of the closed magnetotactic *Desulfobacterota* model strain BW-1 was used as a reference. The region corresponding to the greigite cluster was collapsed. A full analysis with all magnetotactic *Bdellovibrionota* is given in Fig. S6.

Using the MicroScope platform [37] for further comparative genomics analysis, we identified a nearly complete MGC in both FG-1 and FG-2 MAGs including a *mamAB* operon similar to that of *Desulfamplus magnetovallimortis* BW-1 [53], few homologs to *mad* genes such as *mad17, 23*, *25*, *27*, *28*, *29,* and *30* (Fig. 5 and Fig. S6). In addition, atypical two-components systems (TCSs) were predicted near by the MGC in both genomes. The are composed of 5 to 10 contiguous TCSs genes, which is a rare event in bacteria (i.e. <0.2%) according to the P2CS classification [54]. Moreover, both organisms possess at least one complete chemotaxis gene cluster associated with the sensing of an unknown ligand.

We finally explored the potential of this phylum to harbor MTB species by searching for MGCs across all the 772 public genomes available in GTDB R220. We used well annotated *Desulfobacterota* reference genomes such as *Desulfamplus magnetovallimortis* BW-1 [53] and *Solidesulfovibrio magneticus* RS-1 [56], the two MAGs assembled in this study, as well as two *Bdellovibrionota* MAGs previously assembled after magnetic concentration [9], to identify putative homologous magnetosome genes across different genomes. We performed multiple BLASTP searches, using BLOSUM45 matrix and default parameters, for 22 *mam*/*mad* genes annotated in FG MAGs with the expert annotation platform Microscope [37]. A partial or complete MGC with conserved synteny was recovered in 21 *Bdellovibrionota* genomes in addition to those sequenced in this study (Table S2). They are distributed in 4 out of the 11, and three out of the nine *Bdellovibrionota* classes according to the GTDB R220 and R226, respectively (Fig. 3). The genus CAIRTR01 to which FG-2 is affiliated is represented by three MAGs in which a MGC was assembled: i.e. Ki3-D4_bin-355, Kiruna3_bin-3974, and Kiruna_bin-02793 (Fig. 5). These genomes were obtained from the metagenome sequencing of the water column in chemically stratified lakes and ponds in northern landscapes [57]. No close relatives of FG-1 with partial or complete MGC were identified.

The MGC of all *Bdellovibrionota* is structured as that of others deep-branching MTB with a canonical *mamAB* operon like that of BW-1 including few specific *mad* genes of unknown function, and a cluster of *mad* genes, some of which were systematically retrieved, like *mad17*, *mad23*, *mad25*, *mad27*, or *mad30*. No gene cluster coding for greigite was assembled in any of the *Bdellovibrionota* MAGs. When complete or nearly complete, the MGC does not harbor the gene encoding the canonical actin-like protein MamK supposed to polymerize into filaments inside the cell to align magnetosomes as described for instance in the *Desulfobacterota* and *Alphaproteobacteria* models BW-1 and MSR-1 [53,55]. However, *mad28*, another actin-like homolog with a MamK-like fold [58], was found within all MGCs assembled in *Bdellovibrionota* MAGs (Fig. S6). This finding calls into question the use of *mamK* as a universal marker gene for magnetosome formation.

### Genome-wide analysis of Fontaine Goyon metagenome-assembled genomes suggests a predatory lifestyle adapted to low-oxygen and anoxic environments

**Figure 6:**
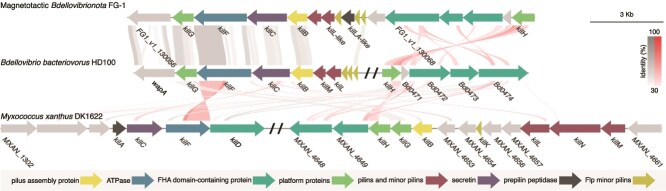
Conservation of the Kil system synteny of the FG-1 genome. The system of the other reference predatory bacteria *Bdellovibrio bacteriovorus* and *myxococcus xanthus* were used as references. Each arrow represents a gene. Annotation was performed using the microscope platform [37], MacSyFinder [62], and custom HMM profiles built previously [63]. Slashes represent mainly truncations and sometimes regions spacing two putative operons. Sequence identities were estimated with BLASTP alignments and are represented by bands, with their intensity reflecting the percentage of identity. Homologous regions in two genomes are connected by grey or red links if they are oriented in the same or opposite direction, respectively. A Kil system like FG-1 is present in the FG-2 genome. The annotated protein sequences of FG-1 are available in Data S3.

We investigated the physiology of FG bacteria through gene content analysis and extended this analysis to the entire phylum to get insight into the lifestyle of magnetotactic *Bdellovibrionota* specifically. Given the fact that representative species of this group are well known for their predatory lifestyle [59], we searched for specific markers associated to predation in *Myxococcota* and *Bdellovibrionota* [60, 61]. We identified a tight adherence (Tad) pilus associated with contact-dependent killing during bacterial predation—homologous to the Kil system previously described in *Myxococcus xanthus*—using both MacSyFinder [62] and custom Hidden Markov Model (HMM) profiles built previously (Fig. 6) [63]. Up to 81% of high-quality *Bdellovibrionota* genomes used here, including FG MAGs (Fig. 3), harbor a Kil system as described in *Bdellovibrio bacteriovorus* HD100 (Table S3) (Fig. 6).

**Figure 7:**
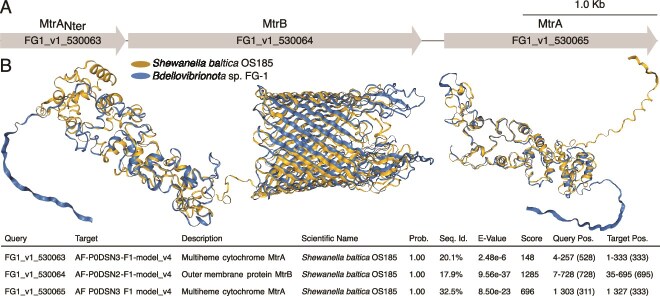
Sequence-structure relationships between the MtrAB operons associated with iron reduction in *Shewanella baltica* and FG-1. (A) Conservation of the operon synteny of the FG-1 genome sequenced in this study. A putative *mtrAB* operon like FG-1 is present in FG-2 genome. (B) AlphaFold structure prediction of FG1_v1_530063, FG1_v1_530064 and FG1_v1_530065 in FG-1 genome aligned with that of MtrA and MtrB proteins in *S. baltica* OS185 identified as the most similar by Foldseek [43] among annotated Swiss-Prot proteins. The annotated protein sequences of FG-1 are available in Data S3.

Energy metabolism of magnetotactic *Bdellovibrionota* was investigated through the Pathway Tools software [39] associated with the BioCyc database collection and the gene function annotation of KofamScan [40] (Table S4 and Table S5). The presence of *ccoNOQP* and *cydAB* genes, coding for a cytochrome *cbb*_3_-type (EC:7.1.1.9) and *bd*-type oxidase (EC:7.1.1.7) respectively, suggests that these organisms are capable of aerobic respiration under low or extremely low O_2_ concentrations [64–66]. Such a *cbb*_3_ oxidase could also have a role in poising proper redox conditions required for magnetite biomineralization as observed in *Magnetospirillum gryphiswaldense* [67]. Both *Bdellovibrionota* MAGs contain also the *hoxFUYH* gene cluster which encodes a NAD^+^-reducing hydrogenase involved in H_2_ oxidation (EC 1.12.1.2). The prediction of a branched-chain amino acid aminotransferase (*ilvE* gene; reaction EC 2.6.1.42) suggests that they are also capable to produce ATP and regenerate NAD^+^ under anaerobic conditions through some oxidative Stickland reactions, which are paired amino acid fermentations [68, 69]. No other alternative terminal electron acceptor was identified using this approach, which is surprising given that FG sediments and the first centimeters of the water column are fully anoxic.

We hypothesized that FG ferruginous sediments could be a source of oxidized iron (III) and iron (II) that some bacteria may use as a pool of terminal electron acceptors or electron donors [70]. We thus looked for remote homologs of iron reductases with the procedure described in the Material and Methods section. Run with defaults settings, FeGenie [41] did not detect any iron related gene in both FG MAGs, not even magnetosomes genes that have been identified in this study (Table S6). Lowering the E-value to 10^−5^, we detected homologs to genes coding for a periplasmic decaheme *c*-type cytochrome MtrA (E-value <10^−64^) and the beta-barrel outer membrane protein MtrB (E-value <10^−45^), which together, link carbon metabolism to the reduction of inorganic substrates in *Shewanella* and *Geobacter* [71] (Table S7). Both genes were also identified with the Microscope platform annotation workflow [37]. The analysis of their structure, confirmed that their closest structural homologs are components of the MtrABC system, which is involved in the dissimilatory reduction of solid metal (hydr)oxides in *Shewanella baltica* OS185 (Fig. 7) [72]. Like *Magnetospirillum magneticum* AMB-1, FG MAGs lack the outer-membrane cytochrome, i.e. nearly always found within the *mtrABC* operon of iron-reducing bacteria [72]. Instead, we identified a gene upstream whose N-terminal region is similar to MtrA. However, as stated previously [41], experimental evidence demonstrated that AMB-1 is an iron-reducing bacterium [73], which indicates that MtrAB may be utilized in iron reduction without the outer-membrane component.

The FG metabolic pathways repertoires show clear evidence for heterotrophy. Maybe because of a better genome assembly quality, FG-1 harbor a broader metabolic repertoire, enabling the degradation of a wide range of organic compounds compared to FG-2 (Table S4). Genes encoding formate dehydrogenase (FDH; EC 1.17.1.9) were detected; however, no other genes associated with the oxidation of C_1_ substrates to formate were identified, such as those involved in the tetrahydromethanopterin (H_4_MPT) pathway or in methanol and methane oxidation. This suggests that the FDH in this organism likely functions in formate detoxification or general redox metabolism rather than in methylotrophic C_1_ utilization. However, it shows evidence for carboxydotrophy (EC 1.2.5.3) as the *coxS* gene coding for the aerobic carbon-monoxide dehydrogenase small subunit was detected [74], but this pathway has never been experimentally observed in *Bdellovibrionota* before.

Finally, some predictions raised questions on the ability of FG bacteria, in particular FG-2, to fix CO_2_ via a variant of the tricarboxylic acid (TCA) cycle (Table S4 and Table S5). In the canonical (oxidative) TCA cycle, the oxoglutarate dehydrogenase complex is a key member that converts 2-oxoglutarate to succinyl-CoA. This multi-enzyme complex typically includes components of the branched-chain α-keto acid dehydrogenase system (EC 1.2.1.25), the pyruvate dehydrogenase system (EC 1.2.1.104), the glycine cleavage system (EC 1.4.1.27), and the acetoin dehydrogenase system (EC 2.3.1.190) [75]. However, none of the genes coding for these enzymes were detected in FG MAGs nor the key enzyme ATP-citrate lyase (EC 2.3.3.8), encoded by *aclAB*. Instead, both MAGs harbored *korAB* genes encoding 2-oxoglutarate synthase (EC 1.2.7.3; K00174–K00175) [76]. This enzyme can substitute for the missing 2-oxoglutarate dehydrogenase complex and allows the cycle to function in either oxidative or reductive modes, depending on the organism’s physiology and environmental conditions [76]. Under anaerobic or microaerophilic conditions with high partial pressures of CO_2_—like those encountered in FG spring—the TCA cycle of magnetotactic *Bdellovibrionota* could operate in reverse to fix CO_2_ and build biomass production [76]. This pathway, along with others, such as reductive monocarboxylic acid cycle or the reductive glycine pathway, was predicted in 10 out of the 52 *Bdellovibrionota* genomes of the class UBA1018 (Table S4).

## Discussion

The most comprehensive view of magnetosome biogenesis has been drawn from the study of cultivated *Alphaproteobacteria* and *Desulfobacterota* models [55,77]. A decade later, metagenomes sequencing of magnetically sorted populations contributed to expand magnetic biomineralization to several bacterial phyla thanks to the detection of the MGC in genome assemblies [9, 78]. Although these findings indicate a much wider taxonomic distribution of magnetosome organelle biogenesis across bacteria than previously thought, our view of MTB functional biodiversity remains incomplete as we do not have cultivated isolates for most phyla. Here, we partially addressed this gap by offering valuable insight into ultrastructure, physiologies, and functions of uncultivated *Bdellovibrionota* species with mini-metagenome sequencing and imaging. No MTB was ever observed in the UBA1018 class previously. Given its predicted metabolism, magnetotaxis, and the bottlenose-shaped morphology, we propose the name *Bdellonasus magneticus* under the Seqcode for the MTB species represented by FG-1, as the representative species of the *Bdellonasaceae* fam nov., *Bdellonasales* ord. nov., and *Bdellonasia* class nov. (see the taxonomy section in Supplementary Results S1). The occurrence of *Bdellonasia* as unique magnetotactic populations in the ferruginous spring, together with their predicted metabolic pathways, provides insights into the factors shaping their ecological niche.

All representative species of *Bdellovibrionota* described to date, such as *Bdellovibrio* spp. or *Bacteriovorax* spp. are aerobic, Gram-negative, curved rods, predatory of other Gram-negative bacteria by penetrating the periplasmic space and dividing in the cytoplasm [79]. Magnetotactic *Bdellonasaceae* seem to use oxygen as well but in hypoxic conditions, which can be surprising given the fact that we found these bacteria exclusively in the anoxic part of the highly reduced sediments. It is unlikely that FG bacteria use sulfate or nitrate as terminal electron acceptors, but we found evidence for the presence of dissimilatory iron(III) reduction in FG MAGs, which would be consistent with the geochemistry of the site. However, the molecular basis of this pathway being poorly known in other bacteria [41], these predictions need to be experimentally validated.

MTB of the FG harbor the same single distinctive flagellum and Kil system described in other close predators [63], which supports these species having the same predatory lifestyle described in other *Bdellovibrionota* previously [45, 80–82]. The flagellum thickness is supposed to ensure greater mechanical stability and torque transmission to generate the propulsion force required for colliding and invading preys at speeds up to 160 μm.s^−1^ [82]. Although the existence of true *Bdellovibrionota* anaerobes was previously unknown [81], our observations and another recent study [83] support that some species may occupy anaerobic niches and possibly predate anaerobic preys. However, some genomic predictions and the geochemistry of the site also challenge the idea that FG bacteria could rely on predation only. Previous observations supported that predation by this group of bacteria is not always essential for their survival [80]. This could also be the case for magnetotactic species, but further research is needed to determine whether the predictions, particularly the involvement of a reductive TCA cycle, are valid.

The *Bdellovibrionota* MAGs obtained in this study and elsewhere after magnetic enrichment were all recovered from freshwater environments only [9]. However, a high-quality MGC was detected in other MAGs obtained without magnetic enrichment. If magnetotaxis was later confirmed in these phyla, it would indicate that magnetotactic *Bdellovibrionota* are not only components of freshwater communities [9, 57, 83], but also some marine habitats. Indeed, a MGC was assembled in genomes of two *Bacteriovoracia* genera recovered from the redoxcline of the Saanich Inlet, British Columbia [84], and that of the Black Sea [85]. Such distribution of magnetotaxis in the tree of *Bdellovibrionota* and across aquatic habitats questions the ecological significance of this group in such oxygen-stratified environments. However, identifying the factors underlying the presence of these bacteria remains challenging due to the complexity of the MTB niche structure and MTB diversity. Here, the advantage of FG is that the extreme geochemistry imposes strong selective pressures, favoring only a limited range of physiological adaptations. The low nitrate, nitrite, sulfate, and oxygen content in the FG spring promoted the growth of bacteria surviving to high CO_2_ and iron content. Whether these conditions are met, at least locally, in other environments could explain the presence of magnetotactic *Bdellovibrionota* in diverse habitats.

At first glance, there does not appear to be a dependency of predation on magnetotaxis and “vice versa”, as not all predatory bacteria display magnetotactic behavior and ~20% of *Bdellovibrionota* MAGs do not encode for a Kil System (Table S3). However, the presence of toxin-antitoxin systems within the MGC in most of the magnetotactic *Bdellonasia* is indicative of a critical role of magnetosome formation in the survival and adaptation of these species. Indeed, we identified homologs of proteins belonging to the RelE/ParE-like or the Phd-Doc families (KO identifiers K19092 and K07341, respectively) (Fig. 5), whose activity is dependent on various stress such as starvation or oxidative stress [86]. For example, in *E. coli*, it would directly reduce translation and assist a stringent response in keeping energy consumption low under starvation conditions [86]. Here, the presence of such system within the MGC could enhance its stabilization within the genome and transmission to offsprings. In addition to helping localize specific chemical species, it is possible that magnetotaxis help to locate specific preyed hosts living at specific depths, and by extension, geochemical niches. Indeed, it was demonstrated that predatory bacteria can recognize specific hosts and preys [87].

Finally, our results question the genetic basis of magnetosome chain formation itself. As mentioned previously, *Bdellovibrionota* from the *Bdellonasia* class (i.e. presently the UBA1018 class in GTDB [33]) form a single chain of spaced and irregularly faceted bullet-shaped magnetosomes in the region of inner positive-cell curvature of the vibrioid cells. Magnetosome chain formation in MTB is supposed to be ensured by MamK, an actin-like protein homolog to MreB, FtsA, or ParM [58, 88]. Several MamK proteins are assembled into dynamic filamentous structures, the magnetoskeleton [89], along the bacterial cytoskeleton to form linear chains of magnetosomes. The correct assembly of magnetosome chains is essential for efficient magnetotaxis. Indeed, in *M. gryphiswaldense* MSR-1, deleting *mamK* results in the formation of magnetosomes clusters instead of chains and reduces the bacteria’s ability to navigate using the Earth’s magnetic field [90]. Similarly, in RS-1, MamK was shown to play a role in chain organization [91]. For this reason, *mamK* is considered as a key marker of magnetotaxis [55]. The gene is used to investigate MTB diversity in metagenomic data [92] and is part of the gene set presumed to be shared by all magnetite-biomineralizing MTB [53]. However, *mamK* is absent from all *Bdellovibrionota* MGCs, which implies another gene fulfills this function without *mamK* in this group. Mad28 could be a good candidate, as this other actin-like protein with a MamK-like fold, is present in most of the magnetotactic *Bdellovibrionota* and other deep-branched taxa of MTB [53, 58]*.* In *mamK* mutant in *M. gryphiswaldense* MSR-1, Mad28 was shown to partially restore magnetosome formation and forms filamentous structures *in vivo*, which was sufficient to create a magnetic moment [58]. In strain RS-1, Mad28 was recently shown to align magnetosomes along the positive curvature during early chain formation [91]. These observations suggest that the genetic control of magnetosome chains formation could be more diverse than currently thought, especially in deep-branching MTB for which little, if no, genetic investigation was performed to depict known and novel magnetosome gene function. *Bdellovibrionota* atypical MGCs and a possible anaerobic lifestyle opens new research directions on the link between magnetotaxis and iron metabolism.

## Supplementary Material

Supplementary_material_ycag116(23)

## Data Availability

Data generated or analyzed during this study are included in this published article and its supporting information files. The 16S rRNA gene amplicon sequences were deposited in the NCBI GenBank database under the accession numbers PV539475-PV539476. The genome assemblies and sequencing data were deposited in the NCBI BioProject database under the accession number PRJNA1064047. Names were deposited under the SeqCode Registry Accession number seqco.de/r:0m3zv347.
