# Supplementary material for: Magnetotactic Bdellovibrionota from a ferruginous spring
Source: ISME Commun. 2026 Apr 24;6(1):ycag116. doi: 10.1093/ismeco/ycag116 (PMC13200544; doi:10.1093/ismeco/ycag116)
Supplement: Supplementary_material_ycag116(23) [file supplementary_material_ycag116(23).zip › Supplementary_material_ycag116/Supplementary_Information.docx]

**Supporting information**

**Magnetotactic *Bdellovibrionota* from a ferruginous spring**

Marine Bergot^1^, Christopher T. Lefevre^1^, Denis S. Grouzdev^2^, Nicolas Menguy^3^, Philippe Ortet^1^, Yann Denis^4^, Eric Viollier^5^, Didier Jézéquel^6,7^ & Caroline L. Monteil^1^*

^1^Aix-Marseille Université, CNRS, CEA, BIAM, UMR7265 Institut de Biosciences and Biotechnologies d’Aix-Marseille, Cadarache research centre, F-13115 Saint-Paul-lez-Durance, France

^2^School of Marine and Atmospheric Sciences, Stony Brook University, Stony Brook, NY, 11794-5000, USA

^3^Sorbonne Université, Muséum National d’Histoire Naturelle, UMR CNRS 7590, Institut de Minéralogie, de Physique des Matériaux et de Cosmochimie (IMPMC), 4 Place Jussieu, 75005 Paris, France

^4^Plateforme Transcriptomique, Aix-Marseille Université, CNRS, IMM - FR3479, Marseille, France

^5^Laboratoire de Chimie Bactérienne (LCB), Institut de Microbiologie de la Méditerranée (IMM), Turing Center for Living Systems, CNRS - Aix-Marseille Université UMR7283, 31 Chemin Joseph Aiguier CS70071, 13402 Marseille Cedex 20, France.

^6^Université Paris Cité, Institut de Physique du Globe de Paris, CNRS, Paris F-75005, France

^7^Laboratoire des Sciences du Climat et de l’Environnement, LSCE–IPSL, CEA–CNRS–UVSQ–Université Paris-Saclay, 91198, Gif-sur-Yvette, France

^8^UMR CARRTEL, INRAE & Université Savoie Mont Blanc, Thonon-les-Bains 74200, France

*Corresponding author. Institut de Biosciences and Biotechnologies d’Aix-Marseille, Cadarache research center, Saint-Paul-lez-Durance F-13115, France

E-mail: [caroline.monteil@cea.fr](mailto:caroline.monteil@cea.fr)

**Contents**

1. **Supplementary Methods**

**Supplementary Methods S1**. Dissolved oxygen concentration measurements

**Supplementary Methods S2**. Procedure for cell sorting

**Supplementary Methods S3**. Assembly binning

**Supplementary Methods S4**. Molecular phylogeny

**Supplementary Methods S5**. Metabolic pathways prediction

**Supplementary Methods S6**. Search for molecular determinants of dissimilatory iron reduction

1. **Supplementary Results**

**Supplementary Results S1**. Protologue

1. **Supplementary Figures**

**Fig. S1**. The sampling site at the Fontaine Goyon spring

**Fig. S2**. Additional transmission electron microscope (TEM) images of the magnetotactic bacteria observed in the FG spring

**Fig. S3**. Fluorescence *in situ* hybridization (FISH) of magnetically collected magnetotactic bacteria from the Fontaine Goyon spring

**Fig****. S4**. Maximum-likelihood tree of the phylum *Bdellovibrionota* showing the distribution of FG MAGs, magnetotactic bacteria and MAGs with an assembled MGC.

**Fig. S5.** Heatmap of the average Amino Acid Identity (AAI) estimated from pairwise genome comparisons of genomes used in the trees of the Figure 4

**Fig. S6.** Conservation of magnetosome gene cluster synteny of the FG MAGs and the other *Bdellovibrionota* MAGs

1. **Supplementary Tables**

**Table S1.** General features of the FG MAGs (Bioproject no. PRJNA1064047)

**Table S2.** List of *Bdellovibrionota* genomes containing a MGC

**Table S3**. List of genomes used in Figure 3 in which at least one kil-homologous gene sequence was detected using the HMM profiles built previously by Herrou et al. [1]

**Table S4.** Comparative analysis of metabolic pathways predicted in at least in one of the *Bdellovibrionota* genomes based on the MetaCyc pathways database.

**Table S5**. KoFamScan output listing genes in FG MAGs with an assigned KEGG Orthology (KO).

**Table S6.** List of homologs to genes related to iron reduction/oxidation in *Bacteria* detected in *Bdellovibrionota* genomes with FeGenie

**Table S7.** List of homologs to genes related to iron reduction/oxidation in *Bacteria* identified in *Bdellovibrionota* genomes with PyHMMER

1. **Supplementary Data**

**Data S1.** Maximum-likelihood tree in newick format of the phylum *Bdellovibrionota* presented in Fig. S4

**Data S2**. Maximum-likelihood tree of the class UBA1018 showing the fine relationships between FG MAGs and high-quality genomes representing each species

**Data S3**. List of protein sequences of FG-1 annotated by the MicroScope platform

1. **References**
2. **Supplementary Methods**

**Supplementary Methods S1**. **Dissolved oxygen concentration measurements**. Dissolved oxygen concentration was measured in the water column and sediments of the microcosms using a fiber-optic oxygen sensor (50-µm tip diameter, REF OXR50) and a FireStingO_2_ meter, both from Pyroscience (Germany). Profiles with a 100 µm resolution, spanning from 20 mm above the sediment to − 25 mm below the sediment were achieved with a Pyroscience MU1 motorized micromanipulator. Sensor calibration was made against saturated humid air and a water solution flushed with N_2 for_ (O_2_ sat. = 100% and 0% respectively). The nature of the sediments heavily loaded with precipitated iron prevented to carry out a cell count profile directly. Their presence/absence in sediment was possible by coring of 1 mL of sediment along with pore water every layer of 2 mm of sediment and to concentrate magnetically the sediment and pore water transferred in an Eppendorf tube.

**Supplementary Methods S2**. **Procedure for cell sorting**. Cell sorting was carried out on sediment samples with an InjectMan® NI2 micromanipulator and a CellTram® vario, hydraulic, manual microinjector from Eppendorf mounted on a Leica DM IL LED microscope equipped with a 63×/0.70 PH objective. The microscope and micromanipulator were placed inside a clean chamber, sterilized beforehand by 1 h germicidal irradiation with ultraviolet (wavelength of the lamp: 254 nm). A 10-µL drop containing magnetically concentrated cells was gently added to a 30-µL drop of filtered environmental water on a hydrophobic coverslip to magnetically transfer magnetotactic cells toward the filtered water. WGA was performed on a sample containing ten and one hundred cells using a sterile microcapillary (TransferTip® (ES), 4 µm inner diameter) into a 4 µL drop of phosphate buffer saline (PBS). This drop was stored at − 20 °C before whole genome amplification.

**Supplementary Methods S3**. **Assembly binning**. The mini-metagenome assembled from the amplified genome of ~100 magnetically sorted cells was processed following the “Anvi'o User Tutorial for Metagenomic Workflow” to visualize them (https://merenlab.org/2016/06/22/anvio-tutorial-v2/) [2]. Contigs longer than 1000 bp were visualized in Anvi’o version 7 [2] and genomes were identified interactively based on tetra-nucleotide frequency, read coverage, GC content and taxonomic profile. Assembly completeness and contamination of the different MAGs were assessed using both CheckM2 [3] via the lineage-specific workflow, and Anvi’o using anvi-estimate-genome-completeness tool with the defaults HMM collections of single-copy core genes named Bacteria_71. Only two bins were retrieved, both of high quality and medium quality, the first having a 16S rRNA gene sequence and a taxonomic assignation identical to that given by one OTU.

**Supplementary Methods S4**. **Molecular phylogeny**. All *Bdellovibrionota* genomes of high quality (i.e*.*, > 90% complete with < 5% redundancy, according to CheckM2 values on GTDB) available in the GTDB Release 220 were downloaded from the GenBank database [4] in January 2025. Genomes were all re-annotated with PROKKA v1.14.6 [5] to homogenize coding sequence predictions. The sequences of the 120 phylogenetically informative markers used by GTDB [6] were then aligned and trimmed using BMGE [7]. For each marker, a tree was constructed using the maximum likelihood method implemented in the IQ-TREE v2.2 software [8,9] and a substitution model selected with ModelFinder [10] with the –MFP option. Then, we used a new method implemented in PhylteR [11] to detect, remove and visualize outliers in the collection of 120 gene trees, *i.e.*, identifying gene trees whose topology do not follow the general trend. Finally, a Maximum-Likelihood tree was built from the concatenated sequences with IQ-TREE [8] and a partition model. Branch robustness was estimated by the ultrafast bootstrapping method implemented in IQ-TREE v2.2 (1000 resamples).

**Supplementary Methods S5**. **Metabolic pathways prediction**. This workflow uses the PathoLogic algorithm of Pathway Tools [39]. First it performs functional annotation with KofamScan v.1.3.0 [40], to link each gene of each genome is to a KO number and functional annotation. Only matches that passed adaptive score thresholds set for that KO family are further used for pathways reconstruction. Then the workflow creates Pathway Tools input files associating enzymatic activities to protein-coding genes thanks to their Enzyme Commission (EC) numbers or MetaCyc reaction identifiers when cross-references between KO, KEGG and MetaCyc identifiers were available. Finally, the obtained Pathway/Genome Databases (PGDB) were queried using the PythonCyc API v2.0.2 (https://github.com/networkbiolab/PythonCyc) to compute pathway completeness rates and pathway presence/absence for each genome and generate a pathway/genome matrix.

**Supplementary Methods S6**. **Search for molecular determinants of dissimilatory iron reduction**. A third approach was applied specifically to search for molecular determinants of dissimilatory iron reduction and oxidation as the diversity of molecular determinants of these pathways in bacteria is still largely unexplored and knowledge is limited to few lineages such as *Geobacterales* or *Enterobacterales* [12], especially in MetaCyc which includes the pathway of *Shewanella* only. This can impede their detection in other distant phyla by some bioinformatic tools as sequence identity might be low. First, we used FeGenie, *i.e.*, a bioinformatics tool that comes with a curated and publicly available database of HMM profiles for enzymes involved in iron acquisition, storage, and redoxcycling in prokaryotes [13]. Then we looked for sequences with remote homology lowering the stringency to define a hit by using the HMM profiles built in FeGenie for iron reduction and oxidation with PyHMMER [14] and a value < 10^-5^ as a minimal threshold for the E-value ignoring the bit score. For any sequence candidate, we then determined the predicted PDB structures of the homologs identified in the FG MAGs using AlphaFold [15]. Then we used Foldseek [16] to compare these structures against the AlphaFold-predicted structures of proteins annotated in the Swiss-Prot database to identify the closest structural matches.

1. **Supplementary Results**

**Supplementary Results S1**. **Protologue**. Names were deposited under the SeqCode Registry Accession number seqco.de/r:0m3zv347.

**Description of *Bdellonasia* class nov.**

*Bdellonasia* (Bdel.lo.na'si.a. N.L. masc. n. *Bdellonasus*, a *Candidatus* genus; –*ia*, ending to denote a class; N.L. neut. pl. n. *Bdellonasia*, the *Bdellonasus* class)

The type genus of the class is *Bdellonasus.*

**Description of *Bdellonasales* ord. nov.**

*Bdellonasales* (Bdel.lo.na.sa'les. N.L. masc. n. *Bdellonasus*, a *Candidatus* genus; –*ales*, ending to denote an order; N.L. fem. pl. n. *Bdellonasales*, the *Bdellonasus* order)

The type genus of the order is *Bdellonasus.*

**Description of *Bdellonasaceae* fam. nov.**

*Bdellonasaceae* (Bdel.lo.na.sa'ce.ae. N.L. masc. n. *Bdellonasus*, a *Candidatus* genus; –*aceae*, ending to denote a family; N.L. fem. pl. n. *Bdellonasaceae*, the *Bdellonasus* family)

The type genus of the family is *Bdellonasus.*

**Description of *Bdellonasus* gen. nov.**

*Bdellonasus* ([Bdel.lo.na](https://protect.checkpoint.com/v2/___http://bdel.lo.na/___.YzJ1OnN0b255YnJvb2s6YzpnOjZiNDI5YzI5N2RjMDJlZDY4NDUwZTk4ODY4MDlmNGYyOjY6ZjRkNDpjZDQ5Y2JmMmVkMzE1MTY4Y2IyZjkwYjI3ODVlNDI0OTM0MDRhMzVlOWE2MjdiZDIyNjkxODRhNGM5ZjY5ZTA5OnA6VDpO)'sus. Gr. fem. n. *bdella*, leech; used here as a reference to the phylum *Bdellovibrionota*, to which the organism belongs; L. masc. n. *nasus*, nose; N.L. masc. n. *Bdellonasus*, a bacterium of *Bdellovibrionota* with a prominent anterior protrusion (“nose”) at the cell tip)

The type species of the genus is *Bdellonasus magneticus.*

**Description of Bdellonasus magneticus sp. nov.**

*Bdellonasus magneticus* ([mag.ne](https://protect.checkpoint.com/v2/___http://mag.ne/___.YzJ1OnN0b255YnJvb2s6YzpnOjZiNDI5YzI5N2RjMDJlZDY4NDUwZTk4ODY4MDlmNGYyOjY6MTVlZDpkYzVmZTI0ZTNkMjAwYmRjYmRjYTFlODg5MWJkZDYwNjQyYjUxOGQzNzU1NzJlNWFlMzZlYjU5YjA4Y2U3NWI0OnA6VDpO)’ti.cus. L. masc. adj. *magneticus*, magnetic; referring to the presence of magnetosomes).

The type genome is GCA_051382295.1.

*Bdellonasus magneticus* cells are vibrioid with an approximative length and width of 1.45 µm and 0.40 µm, respectively. They produce a single polar ⁓53.2 nm thick flagellum and are characterized by the presence of a 200-300 nm-long a sharp, asymmetrical and elongated anterior pole, bottlenose-shaped. Cells are magnetotactic and produce a single chain of approximately 5 bullet-shaped magnetite particles ~70 nm long. The genome of this species possesses a gene coding for an aerobic carbon monoxide dehydrogenase, a formate dehydrogenase, and *bd* and *cbb_3_*-type cytochrome *c* oxidases—involved in aerobic respiration under low O_2_ conditions—as well as putative genes for iron reduction. The genome harbors genes for heterotrophy and a Kil system involved in predation.

1. **Supplementary Figures**


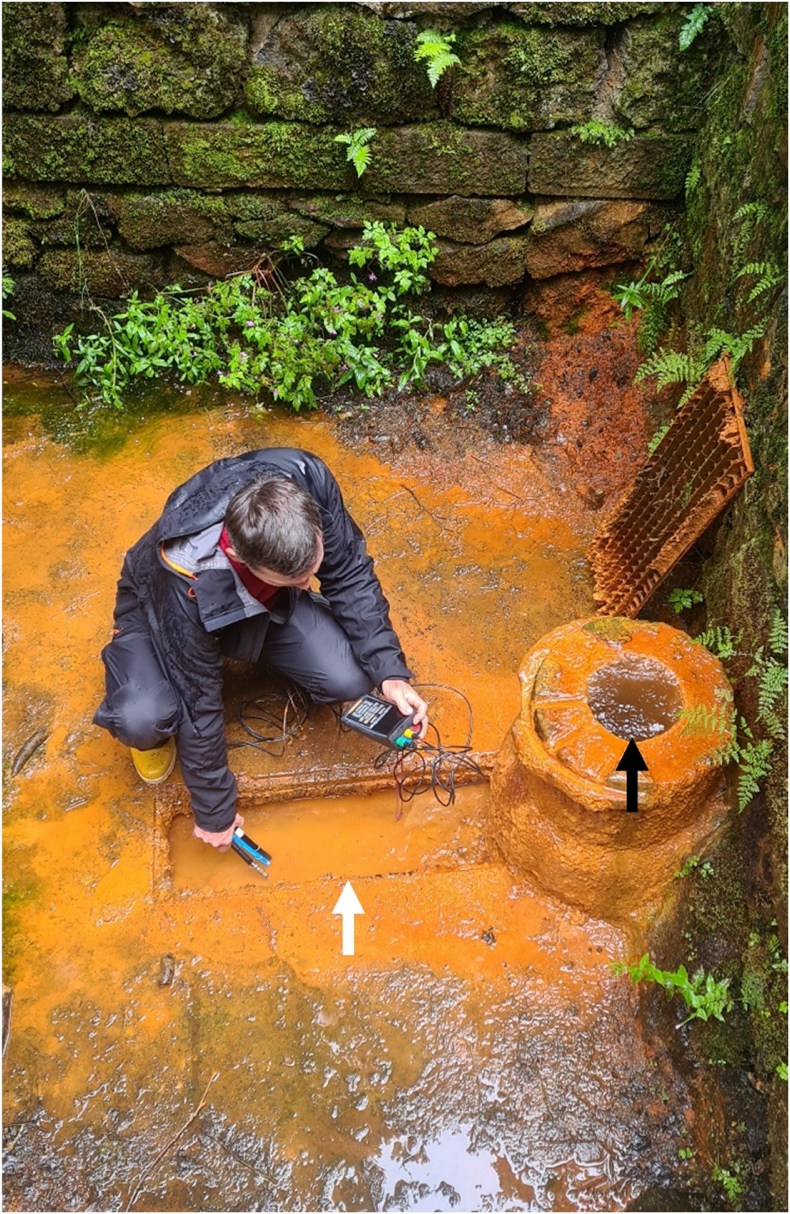
**Figure S1.** **The sampling site at the Fontaine Goyon spring.** Black arrow shows the opening of the spring from a built concrete structure. The water overflow naturally, the concrete structure was built in the 19^th^ century in an attempt to commercialize the water from this spring. The red color is an indication of an iron rich water that oxidized and produce rust once in contact with oxygen. The white arrow indicates the channel where the water from the spring flows and stabilizes. This channel is approximately 20 cm deep and is filled with about 10 cm of sediment where magnetotactic bacteria thrive. All geochemical analyses and samples were done in this channel as shown in this image in which D. Jézéquel is measuring the temperature, dissolved oxygen, conductivity and pH of the water of the channel.

**Figure S2.** **Additional** **transmission electron microscope (TEM) images of the magnetotactic bacteria observed in the FG spring.**

**Figure S3.** **Fluorescence *in situ* hybridization (FISH) of magnetically collected magnetotactic bacteria from the Fontaine Goyon spring**. It was possible to obtain transmitted light confocal microscope images (left panel), and laser scan images of cells 1) labeled with 4',6-diamidino-2-phenylindole (DAPI), 2) hybridized with a bacteria-specific probe (Eubp), and finally 3) hybridized with the specific ATTO488-labeled probe FGp (5'- TTGCGCTTTCGCTTCCCTCTGTACCGACCA -3', complementary to nucleotides close to the 1243 bp region of the 16S rRNA molecule). White arrows indicate the small vibrioid cells that could be seen under the transmission mode of the confocal. The large rod bacterium is used as a negative control as the specific FG probe did not hybridize with its 16S rRNA gene sequence.

**Figure S4**. **Maximum-likelihood tree of the phylum *Bdellovibrionota* showing the distribution of FG MAGs, magnetotactic bacteria and MAGs with an assembled MGC.** The tree was drawn as described in the Material and Methods section based on the conserved markers used in the GTDB [6]. We selected a set of genomes of high quality (*i.e.*, > 90% complete with < 5% redundancy according to CheckM2 [3] representing each genus (GTDB R220). The tree was rooted with representative members of the phylum *Nitrospirota*. Branch lengths represent the number of substitutions per site. The values next to the internal nodes represent the statistical support (ultra-fast bootstrapping approach 1000 replicates). The corresponding GenBank accession numbers are given in the sequence names, along with the corresponding order "o_", family "f_", genus "g_" and species "s_" names in GTDB release 220 [6] (<https://gtdb.ecogenomic.org>). In the GTDB release 226, the phylum *Bdellovibrionota* is split into two, with the *Oligoflexia* and UBA2361 classes being reassigned into an undescribed phylum: the phylum Bdellovibrionota_B. The red and blue colored leaves represent GTDB genera in which at least one genome was obtained from a metagenome of magnetically sorted cells, or from a metagenome without magnetic purification, respectively. The newick file is available as **Data S1**.

**Figure S5**. **Heatmap of the average Amino Acid Identity (AAI %) estimated from pairwise genome comparisons of genomes used in the trees of the Fig. 4.**

**
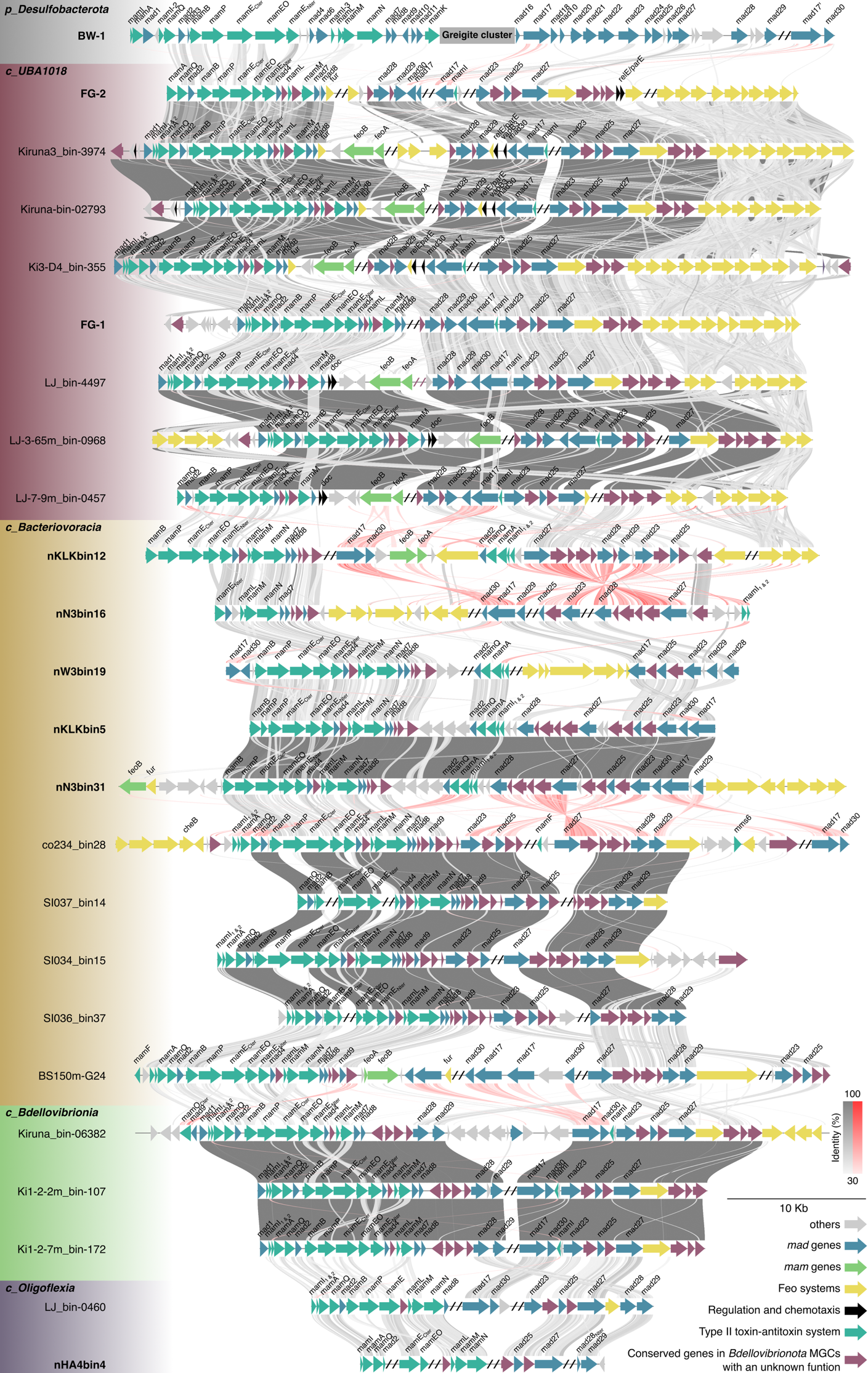
Figure S6.** **Conservation of magnetosome gene cluster synteny of the FG MAGs and the other *Bdellovibrionota* MAGs.** Names in bold represent the genomes obtained from a magnetic pellet. Names in regular font represent MAG from stratified environments (referenced in **Table S2**). Each arrow represents a gene. Homologous families were then determined by the presence of conserved domains using the Microscope platform [17]. The annotated magnetosome genes described in the reference genomes of *Magnetospirillum gryphiswaldense* MSR-1 [18] and *Desulfamplus magnetovallimortis* BW-1 [19] are colored in turquoise and blue (i.e., *mam* and *mad* genes, respectively). Genes of unknown function or not conserved in MTB are colored in grey whereas those conserved in magnetotactic *Bdellovibrionota* only, are colored in wine red. Slashes represent mainly truncations and sometimes regions spacing two putative operons. Sequence identities were estimated with BLASTP alignments and are represented by bands, with their intensity reflecting the percentage of identity. Homologous regions in two genomes are connected by grey or red links if they are oriented in the same or opposite direction, respectively. The MGC of the closed magnetotactic *Desulfobacterota* model strain BW-1 was used as a reference. The region corresponding to the greigite cluster was collapsed. The *Oligoflexia* class is affiliated to a novel phylum *Bdellovibrionota*_B in the GTDB R226 that has no official status in the List of Prokaryotic names with Standing in Nomenclature.

1. **Supplementary Tables**

**Table S1.** General features of the FG MAGs (Bioproject no. PRJNA1064047)

|  |  |  |  |
| --- | --- | --- | --- |
|  |  |  |  |
| MAG name | | FG-1 | FG-2 |
| Size |  | 4459092 | 4381748 |
|  | Contig number | 158 | 433 |
|  | GC content | 51.29% | 49.79% |
|  | Completion | 97.60% | 84.80% |
|  | Contamination | 4.91% | 4.41% |
| Predicted number of ORFs | | 4474 | 4754 |
|  | Coding sequences (CDS) | 4411 | 4694 |
|  | Coding potential | 91.25% | 90.15% |
|  | Average CDS length | 928.61 bp | 841.9 bp |
| tRNA genes | | 46 | 39 |
| rRNA operons | | 1 | 0 |
|  |  |  |  |
| Macromolecular systems* | |  |  |
|  | Flagellum | 1 | 0 |
|  | Tad (Kil-like) | 1 | 1 |
|  | MOB | 6 | 7 |
|  | dCONJ_typeT | 2 | 0 |
|  | T2SS | 1 | 0 |
| Defense systems** | |  |  |
|  | Restriction Modification (RM) | 1 | 1 |
|  | AbiL | 0 | 1 |
|  | AbiE | 2 | 2 |
|  | MazEF | 1 | 1 |
|  | Rst_3HP | 1 | 0 |
|  | Bacteriophage Exclusion | 1 | 0 |
|  | RloC | 1 | 0 |
|  | Tiamat | 1 | 0 |
|  |  |  |  |
| CDS classification based on COG categories | |  |  |
|  | D \| Cell cycle control, cell division, chromosome partitioning | 1.81% | 1.55% |
|  | M \| Cell wall/membrane/envelope biogenesis | 4.64% | 5.50% |
|  | N \| Cell motility | 2.40% | 2.46% |
|  | O \| Posttranslational modification, protein turnover, chaperones | 2.80% | 2.55% |
|  | T \| Signal transduction mechanisms | 5.27% | 5.28% |
|  | U \| Intracellular trafficking, secretion, and vesicular transport | 3.08% | 2.67% |
|  | V \| Defense mechanisms | 1.27% | 1.36% |
|  | W \| Extracellular structures | 0.00% | 0.02% |
|  | Z \| Cytoskeleton | 0.32% | 0.13% |
|  | A \| RNA processing and modification | 0.02% | 0.06% |
|  | B \| Chromatin structure and dynamics | 0.07% | 0.06% |
|  | J \| Translation, ribosomal structure and biogenesis | 4.18% | 3.86% |
|  | K \| Transcription | 5.02% | 4.50% |
|  | L \| Replication, recombination and repair | 4.98% | 6.09% |
|  | C \| Energy production and conversion | 4.73% | 4.69% |
|  | E \| Amino acid transport and metabolism | 3.55% | 3.52% |
|  | F \| Nucleotide transport and metabolism | 1.97% | 2.10% |
|  | G \| Carbohydrate transport and metabolism | 2.26% | 2.78% |
|  | H \| Coenzyme transport and metabolism | 2.15% | 2.36% |
|  | I \| Lipid transport and metabolism | 2.08% | 2.42% |
|  | P \| Inorganic ion transport and metabolism | 2.06% | 2.19% |
|  | Q \| Secondary metabolites biosynthesis, transport and catabolism | 1.11% | 1.02% |
|  | S \| Function unknown | 16.94% | 15.75% |
|  |  |  |  |
| *Maromolecular systems were annotated using MacSyFinder version 2.1.2. **Defense systems were annotated using DefenseFinder version 1.2.2. ***Classification based on COG categories using eggNOG version 5.0.2 and eggNOG-mapper version 2.1.12. | | | |

**Table S2.** **List of *Bdellovibrionota* genomes containing a MGC.** Only MAGs from Lin et al. (2020) and this study were obtained from a magnetic pellet.

|  |  |  |  |  |  |  | |
| --- | --- | --- | --- | --- | --- | --- | --- |
| **Species*** | | **Accession number** | **CheckM2 completion** | **CheckM2 redundancy** | **Taxonomy*** | **MAG name** | |
|  |  |  |  |  |  |  | |
| Sequenced in Buck *et al*. (2021) https://doi.org/10.1038/s41597-021-00910-1 | | | | | | | |
|  |  |  |  |  |  |  | |
|  | s__CAILAO01 sp903832105 | GCA_903832105.1 | 87.98 | 7.26 | p__Bdellovibrionota;c__Oligoflexia;o__Oligoflexales;f__RGVZ01 | LJ_bin-0460 | |
|  | s__CAIRTR01 sp903881565 | GCA_903918395.1 | 95.4 | 4.37 | p__Bdellovibrionota;c__UBA1018;o__UBA1018;f__UBA1018 | Ki3-D4_bin-355 | |
|  | s__CAIRTR01 sp903881565 | GCA_903881565.1 | 98.75 | 2.68 | p__Bdellovibrionota;c__UBA1018;o__UBA1018;f__UBA1018 | Kiruna3_bin-3974 | |
|  | s__CAIRTR01 sp903881565 | GCA_903853655.1 | 98.97 | 3.8 | p__Bdellovibrionota;c__UBA1018;o__UBA1018;f__UBA1018 | Kiruna_bin-02793 | |
|  | s__CAITVN01 sp903895855 | GCA_903879355.1 | 89.4 | 4.39 | p__Bdellovibrionota;c__UBA1018;o__UBA1018;f__UBA1018 | LJ-3-65m_bin-0968 | |
|  | s__CAITVN01 sp903895855 | GCA_903895855.1 | 93.16 | 2.06 | p__Bdellovibrionota;c__UBA1018;o__UBA1018;f__UBA1018 | LJ_bin-4497 | |
|  | s__CAITVN01 sp903895855 | GCA_903840645.1 | 83.98 | 4.61 | p__Bdellovibrionota;c__UBA1018;o__UBA1018;f__UBA1018 | LJ-7-9m_bin-0457 | |
|  | s__CAIYID01 sp903926205 | GCA_903843195.1 | 99.96 | 1.09 | p__Bdellovibrionota;c__Bdellovibrionia;o__Bdellovibrionales;f__Bdellovibrionaceae | Ki1-2-2m_bin-107 | |
|  | s__CAIYID01 sp903926205 | GCA_903897095.1 | 98.46 | 1.78 | p__Bdellovibrionota;c__Bdellovibrionia;o__Bdellovibrionales;f__Bdellovibrionaceae | Ki1-2-7m_bin-172 | |
|  | s__CAIYID01 sp903926205 | GCA_903926205.1 | 99.96 | 1.13 | p__Bdellovibrionota;c__Bdellovibrionia;o__Bdellovibrionales;f__Bdellovibrionaceae | Kiruna_bin-06382 | |
|  |  |  |  |  |  |  | |
| Sequenced in Lin *et al.* (2021) https://doi.org/10.1038/s41396-020-00889-4 | | | | | | | |
|  |  |  |  |  |  |  | |
|  | s__JABGUH01 sp018646675 | GCA_018646675.1 | 94.42 | 1.36 | p__Bdellovibrionota;c__Bacteriovoracia;o__Bacteriovoracales;f__Bacteriovoracaceae | SI034_bin15 | |
|  | s__JABGUH01 sp018646675 | GCA_018674685.1 | 81.94 | 2.61 | p__Bdellovibrionota;c__Bacteriovoracia;o__Bacteriovoracales;f__Bacteriovoracaceae | SI036_bin37 | |
|  | s__JABGUH01 sp018646675 | GCA_018672895.1 | 82.98 | 2.05 | p__Bdellovibrionota;c__Bacteriovoracia;o__Bacteriovoracales;f__Bacteriovoracaceae | SI037_bin14 | |
|  | s__JABGUH01 sp018646675 | GCA_018645025.1 | 94.97 | 0.76 | p__Bdellovibrionota;c__Bacteriovoracia;o__Bacteriovoracales;f__Bacteriovoracaceae | co234_bin28 | |
|  |  |  |  |  |  |  | |
| Sequenced in Lin *et al.* (2020) https://doi.org/10.1186/s40168-020-00931-9 | | | | | | | |
|  |  |  |  |  |  |  | |
|  | s__JADFZC01 sp015232735 | GCA_015232735.1 | 56.03 | 4.68 | p__Bdellovibrionota;c__Oligoflexia;o__Oligoflexales;f__Oligoflexaceae | nHA4bin4 | |
|  | s__JADFZW01 sp015232355 | GCA_015232355.1 | 94.55 | 3.2 | p__Bdellovibrionota;c__Bacteriovoracia;o__Bacteriovoracales;f__Bacteriovoracaceae | nKLKbin12 | |
|  | s__JADFZW01 sp015232015 | GCA_015232015.1 | 84.73 | 2.61 | p__Bdellovibrionota;c__Bacteriovoracia;o__Bacteriovoracales;f__Bacteriovoracaceae | nN3bin16 | |
|  | s__JADGAN01 sp015231955 | GCA_015232255.1 | 85.04 | 2 | p__Bdellovibrionota;c__Bacteriovoracia;o__Bacteriovoracales;f__Bacteriovoracaceae | nKLKbin5 | |
|  | s__JADGAN01 sp015231955 | GCA_015231955.1 | 95.21 | 3.04 | p__Bdellovibrionota;c__Bacteriovoracia;o__Bacteriovoracales;f__Bacteriovoracaceae | nN3bin31 | |
|  | s__JADGAN01 sp015231325 | GCA_015231325.1 | 65.98 | 3.18 | p__Bdellovibrionota;c__Bacteriovoracia;o__Bacteriovoracales;f__Bacteriovoracaceae | nW3bin19 | |
|  |  |  |  |  |  |  | |
| Sequenced in Cabello-Yeves *et al.* (2021) https://doi. org/10.1186/s40793-021-00374-1 | | | | | | | |
|  |  |  |  |  |  |  | |
|  | s__JADHTQ01 sp016784965 | GCA_016784965.1 | 97.47 | 3.78 | p__Bdellovibrionota;c__Bacteriovoracia;o__Bacteriovoracales;f__Bacteriovoracaceae | BS150m-G24 | |
|  |  |  |  |  |  |  | |
| *Sequenced in this study* | | | | | | | |
|  |  |  |  |  |  |  | |
|  | ***Bdellonasus magneticus*** | **GCA_051382295.1** | **97.6** | **4.91** | **p__Bdellovibrionota;c__UBA1018;o__UBA1018;f__UBA1018** | **FG-1** | |
|  | **g__CAIRTR01** | **GCA_051382305.1** | **84.8** | **4.41** | **p__Bdellovibrionota;c__UBA1018;o__UBA1018;f__UBA1018;g__CAIRTR01** | **FG-2** | |
|  |  |  |  |  |  | |  |
| *according to GTDB R220 | |  |  |  |  | |  |

**Table S3.** **List of genomes and MAGs from Figure 3 in which at least one *kil*-homologous gene sequence was detected using the HMM profiles built previously by Herrou et al. [1].** Each genome is associated to its accession number in NCBI and its taxonomy according to GTDB R220 (d__domain; p__phylum; c__class; o__order; f__family; g__genus; s__species) (NCBI RefSeq database, February 2025). Only best hits with a minimal E-value of 10E^−25^ are reported. The index of their genomic coordinates after a reannotation with PROKKA and the accession number in the corresponding genome are given, as well as the exact E-value. Indexes enable to identify gene synteny.

See the Supplementary Table file: Table S3 (XLSX).

**Table S4.** **Comparative analysis of metabolic pathways predicted in at least in one of the *Bdellovibrionota* genomes based on the MetaCyc pathways database**. MetaCyc pathways are supported by the Pathway tools software developed by Peter Karp and his team at SRI international [20]. These pathways were predicted using the PathoLogic module which computes an initial set of pathways by comparing a genome annotation to the metabolic reference database MetaCyc as described previously [21]. Green and white boxes represent "predicted" and "not predicted" pathways. They do not reflect a pathway completion rate. Absence of prediction can be linked to the absence of a single reaction/enzyme/gene judged mandatory for the pathway realization. Yet this absence can be a false negative and be linked to the quality of the draft genome assembly.

See the Supplementary Table file: Table S4 (XLSX).

**Table S5. KoFamScan output listing genes in FG MAGs with an assigned KEGG Orthology (KO).** Each gene name from the fasta file annotated with PROKKA is linked to a KO number and functional annotation, a HMM E-value / Score. Only matches that passed adaptive score thresholds set for that KO family are shown. The KO identifiers can be mapped to KEGG pathway maps, BRITE hierarchies, and modules. Only matches that passed adaptive score thresholds were used for pathways reconstruction.

See the Supplementary Table file: Table S5 (XLSX).

**Table S6.** **List of homologs to genes related to iron reduction/oxidation in *Bacteria* detected in 772 *Bdellovibrionota* genomes from the GTDB release 220 with FeGenie [13].**

See the Supplementary Table file: Table S6 (XLSX).

**Table S7.** **List of homologs to genes related to iron reduction/oxidation in *Bacteria* identified in 206 *Bdellovibrionota* genomes used in Figure 3**. The research was performed using the HMM profiles built in FeGenie for iron reduction and oxidation with PyHMMER and a E-value < 10^-5^ as a minimal threshold. Description of the genes and classification are given in Garber et al. [13]. When the analysis returns a hit, it gives an gene accession number given by the de novo annotation with Prokka [5] and a corresponding E-value.

See the Supplementary Table file: Table S7 (XLSX).

1. **Supplementary Data**

**Data S1**. **Maximum-likelihood tree in newick format of the phylum *Bdellovibrionota* presented in Figure S4.**

See the Supplementary text file (Newick format): Data S1 (.TXT).

**Data S2**. **Maximum-likelihood tree of the class UBA1018 showing the fine relationships between FG MAGs and high-quality genomes representing each species.** The tree was drawn as described in the Material and Methods section based on 120 conserved bacterial markers used for the GTDB. We selected a set of genomes of good quality (i.e*.*, > 90% complete with < 5% redundancy according to CheckM2 [3] representing each genus (February 2025). The tree was rooted with representative members of the phylum *Bacteriovoracia*. Branch lengths represent the number of substitutions per site. The values next to the internal nodes represent the statistical support (ultra-fast bootstrapping approach 1000 replicates). The corresponding GenBank accession numbers are given in the sequence names, along with the corresponding order "o_", family "f_", genus "g_" and species "s_" names in GTDB [6] (<https://gtdb.ecogenomic.org>).

See the Supplementary text file (Newick format): Data S2 (.TXT).

**Data S3**. **List of protein sequences of FG-1 annotated by the microscope platform.**

See the Supplementary text file (Fasta format): Data S3 (.TXT).

1. **References**

1. Herrou J, My L, Monteil CL, Bergot M, Jain R, Martinez E *et al.* Tad pili with adaptable tips mediate contact-dependent killing during bacterial predation. *Nat Commun* 2025;**16**(1):4425. https://doi.org/10.1038/s41467-025-58967-0.

2. Eren AM, Kiefl E, Shaiber A, Veseli I, Miller SE, Schechter MS *et al.* Community-led, integrated, reproducible multi-omics with anvi’o. *Nat Microbiol* 2021;**6**(1):3–6. https://doi.org/10.1038/s41564-020-00834-3.

3. Chklovski A, Parks DH, Woodcroft BJ, Tyson GW. CheckM2: a rapid, scalable and accurate tool for assessing microbial genome quality using machine learning. *Nat Methods* 2023;**20**(8):1203–12. https://doi.org/10.1038/s41592-023-01940-w.

4. Benson DA, Cavanaugh M, Clark K, Karsch-Mizrachi I, Lipman DJ, Ostell J *et al.* GenBank. *Nucleic Acids Res* 2013;**41**(Database issue):D36-42. https://doi.org/10.1093/nar/gks1195.

5. Seemann T. Prokka: rapid prokaryotic genome annotation. *Bioinformatics* 2014;**30**(14):2068–9. https://doi.org/10.1093/bioinformatics/btu153.

6. Parks DH, Chuvochina M, Rinke C, Mussig AJ, Chaumeil PA, Hugenholtz P. GTDB: an ongoing census of bacterial and archaeal diversity through a phylogenetically consistent, rank normalized and complete genome-based taxonomy. *Nucleic Acids Res* 2022;**50**(D1):D785–94. https://doi.org/10.1093/nar/gkab776.

7. Criscuolo A, Gribaldo S. BMGE (Block Mapping and Gathering with Entropy): a new software for selection of phylogenetic informative regions from multiple sequence alignments. *BMC Evol Biol* 2010;**10**:210. https://doi.org/10.1186/1471-2148-10-210.

8. Minh BQ, Schmidt HA, Chernomor O, Schrempf D, Woodhams MD, Haeseler A von *et al.* IQ-TREE 2: new models and efficient methods for phylogenetic inference in the genomic era. *Mol Biol Evol* 2020;**37**(5):1530–4. https://doi.org/10.1093/molbev/msaa015.

9. Nguyen LT, Schmidt HA, Haeseler A von, Minh BQ. IQ-TREE: a fast and effective stochastic algorithm for estimating maximum-likelihood phylogenies. *Mol Biol Evol* 2015;**32**(1):268–74. https://doi.org/10.1093/molbev/msu300.

10. Kalyaanamoorthy S, Minh BQ, Wong TKF, Haeseler A von, Jermiin LS. ModelFinder: fast model selection for accurate phylogenetic estimates. *Nat Methods* 2017;**14**(6):587–9. https://doi.org/10.1038/nmeth.4285.

11. Comte A, Tricou T, Tannier E, Joseph J, Siberchicot A, Penel S *et al.* PhylteR: efficient identification of outlier sequences in phylogenomic datasets. *Mol Biol Evol* 2023;**40**(11):msad234. https://doi.org/10.1093/molbev/msad234.

12. Shi L, Squier TC, Zachara JM, Fredrickson JK. Respiration of metal (hydr)oxides by *Shewanella* and *Geobacter*: a key role for multihaem *c*-type cytochromes. *Mol Microbiol* 2007;**65**(1):12–20. https://doi.org/10.1111/j.1365-2958.2007.05783.x.

13. Garber AI, Nealson KH, Okamoto A, McAllister SM, Chan CS, Barco RA *et al.* FeGenie: a comprehensive tool for the identification of iron genes and iron gene neighborhoods in genome and metagenome assemblies. *Front Microbiol* 2020;**11**:37. https://doi.org/10.3389/fmicb.2020.00037.

14. Larralde M, Zeller G. PyHMMER: a Python library binding to HMMER for efficient sequence analysis. *Bioinformatics* 2023;**39**(5):btad214. https://doi.org/10.1093/bioinformatics/btad214.

15. Jumper J, Evans R, Pritzel A, Green T, Figurnov M, Ronneberger O *et al.* Highly accurate protein structure prediction with AlphaFold. *Nature* 2021:1–11. https://doi.org/10.1038/s41586-021-03819-2.

16. Kempen M van, Kim SS, Tumescheit C, Mirdita M, Lee J, Gilchrist CLM *et al.* Fast and accurate protein structure search with Foldseek. *Nat Biotechnol* 2024;**42**(2):243–6. https://doi.org/10.1038/s41587-023-01773-0.

17. Vallenet D, Calteau A, Dubois M, Amours P, Bazin A, Beuvin M *et al.* MicroScope: an integrated platform for the annotation and exploration of microbial gene functions through genomic, pangenomic and metabolic comparative analysis. *Nucleic Acids Res* 2020;**48**(D1):D579–89. https://doi.org/10.1093/nar/gkz926.

18. Uebe R, Schüler D. Magnetosome biogenesis in magnetotactic bacteria. *Nat Rev Microbiol* 2016;**14**(10):621–37. https://doi.org/10.1038/nrmicro.2016.99.

19. Lefèvre CT, Trubitsyn D, Abreu F, Kolinko S, Jogler C, Almeida LGP de *et al.* Comparative genomic analysis of magnetotactic bacteria from the *Deltaproteobacteria* provides new insights into magnetite and greigite magnetosome genes required for magnetotaxis. *Environ Microbiol* 2013;**15**:2712–35. https://doi.org/10.1111/1462-2920.12128.

20. Caspi R, Billington R, Keseler IM, Kothari A, Krummenacker M, Midford PE *et al.* The MetaCyc database of metabolic pathways and enzymes - a 2019 update. *Nucleic Acids Res* 2020;**48**(D1):D445–53. https://doi.org/10.1093/nar/gkz862.

21. Mangin CC, Benzerara K, Bergot M, Menguy N, Alonso B, Fouteau S *et al.* Magnetotactic bacteria affiliated with diverse *Pseudomonadota* families biomineralize intracellular Ca-carbonate. *ISME J* 2025;**19**(1):wrae260. https://doi.org/10.1093/ismejo/wrae260.
