# Supplementary figures and images for: Magnetotactic Bdellovibrionota from a ferruginous spring
Source: ISME Commun. 2026 Apr 24;6(1):ycag116. doi: 10.1093/ismeco/ycag116 (PMC13200544; doi:10.1093/ismeco/ycag116)

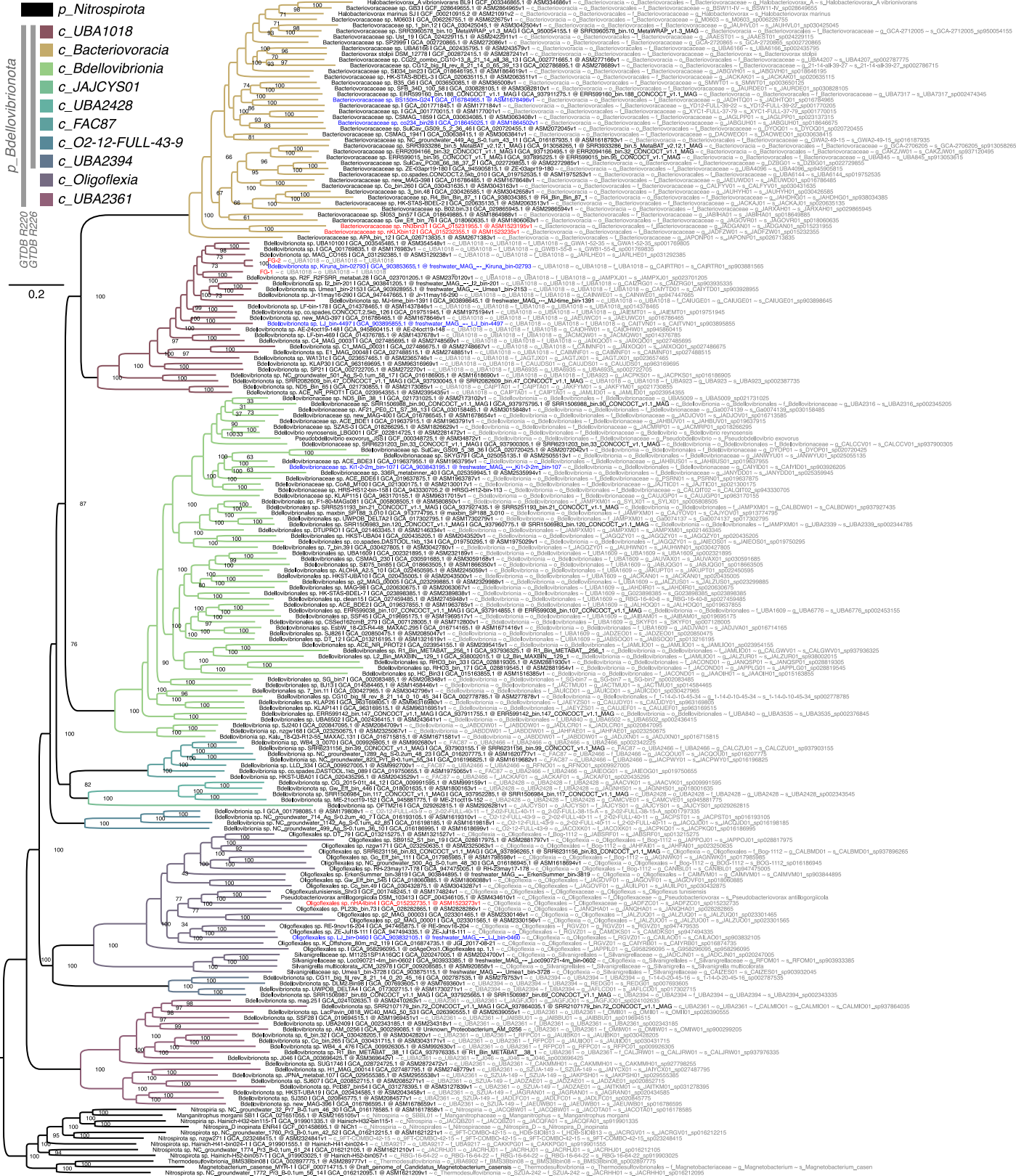

Supplement: Supplementary_material_ycag116(23) [file supplementary_material_ycag116(23).zip › Supplementary_material_ycag116/Figure_S4_Full_tree.pdf]
